# Factors Affecting Haul-Out Behavior of Harbor Seals (*Phoca vitulina*) in Tidewater Glacier Inlets in Alaska: Can Tourism Vessels and Seals Coexist?

**DOI:** 10.1371/journal.pone.0125486

**Published:** 2015-05-27

**Authors:** Gail M. Blundell, Grey W. Pendleton

**Affiliations:** Alaska Department of Fish and Game, Division of Wildlife Conservation, Juneau, Alaska, United States of America; Sonoma State University, UNITED STATES

## Abstract

Large numbers of harbor seals (*Phoca vitulina*) use habitat in tidewater glaciers in Alaska for pupping, breeding, and molting. Glacial fjords are also popular tourist destinations; however, visitation by numerous vessels can result in disturbance of seals during critical life-history phases. We explored factors affecting haul-out behavior of harbor seals at a glacial site frequented by tourism vessels. In 2008-10, we deployed VHF transmitters on 107 seals in Endicott Arm, Alaska. We remotely monitored presence and haul-out behavior of tagged seals and documented vessel presence with time-lapse cameras. We evaluated the influence of environmental and physical factors on the probability of being hauled out, duration of haul-out bouts, and as factors associated with the start and end of a haulout. Location, season, hour, and interactions of location by year, season, hour, and sex significantly influenced haul-out probability, as did ice, weather, and vessels. Seals were more likely to be hauled out with greater ice availability during the middle of the day, and less likely to be hauled out if vessels were present. Cruise ships had the strongest negative effect; however, most vessel types negatively affected haul-out probability. Haul-out duration was longest in association with starting on incoming tides, clear skies, no precipitation, occurring in the middle of the day, and ending in the late afternoon or evening. End of haulouts was associated with increasing cloud cover, low ice availability, and vessel presence; large-sized tourism vessels or all-vessel-types combined were significant predictors of ending a haul-out bout. Probability of being hauled out was highest in June, during pupping season. Potential disturbances of harbor seals could be reduced, enabling longer resting times for seals and fewer interruptions for nursing pups, if vessels focused the majority of visits to glacial habitat to before or after the hours of 08:00-17:00 or, less optimally, 09:00-16:00.

## Introduction

Scenic fjords filled with icebergs calved from tidewater glaciers are popular tourism destinations in Alaska [1–4]. Many of those sites are also important habitat for pupping, breeding, and molting harbor seals (*Phoca vitulina richardii*), seasonally supporting disproportionately large numbers of mothers and pups relative to the total number of seals in the area [5, 6]. The pronounced pattern of seasonal use [7], along with movement [8] and genetic data [9] indicate that while many seals may overwinter elsewhere, most return to glacial habitat to give birth and breed. The long-term viability of seals using glacial habitat may be at risk due to the rapid loss of tidewater glaciers as a result of climate change [10–12] and the growing importance of glacial habitat in Alaska as a tourism destination [1, 2, 4].

Activities of tourism vessels have been shown to negatively affect marine mammals. Noise generated by vessels may result in displacement or avoidance [13], increased stress, changes in swim-speed, foraging and diving behavior, and stranding of marine mammals (studies reviewed by [14]), and may also disrupt communication [15]. In a study of bottlenose dolphins (*Tursiops spp*.), the presence of vessels resulted in a decrease in resting, foraging, and socializing, and an increase in traveling [16]. Dolphins with calves increased erratic activity and movements as vessel traffic and tourism activities increased [17]. In another study of bottlenose dolphins, a decline in numbers was documented in association with an increase in vessel traffic [18]. Common dolphins (*Delphinus spp*) disrupted foraging and resting bouts [19] in the presence of vessels, while humpback whales (*Megaptera novaeangliae)* spent more time submerged, and pods with calves were more sensitive to vessel presence than those without calves [20].

In harbor seals, the types, numbers, and activity of vessels, vessel distance and approach pathway, and whether seals had pups or not, all resulted in varying disturbance responses (i.e., flushing from a haulout) at different locations. More vessels present resulted in more disturbance [21, 22], as did vessels approaching directly [1, 23] or moving erratically, including starting and stopping or moving at variable speeds [21, 22]. Seals with pups were more sensitive to vessel presence than those without [22, 24], and seals were less likely to flush during molt than at other times [22]. Harbor seals alerted to vessel presence at >800m [22], responded with a flight response at distances of 510-830m for a directly approaching 10m vessel [25], and in glacial habitat, cruise ships could result in disturbances at distances ≥500m [1, 3]. In some studies the type of vessel did not affect flushing distance; while some seals flushed at distances >200m [22, 24] there was no difference in percent of seals that flushed at >200m vs. 100-200m[22], but more seals flushed at <100m [1, 22, 23]. Some studies reported that harbor seals were more likely to flush at the approach of kayaks or canoes [2, 22, 24] or stopped motor vessels, even at distances >91m [21, 23]. Conversely, Young et al. [3] noted that motorized vessels, especially cruise ships, disturbed greater numbers of seals in glacial habitat than did kayaks, and Henry and Hamill [22] found that harbor seals at terrestrial haulouts alerted sooner to motorized vessels than to kayaks.

Although numerous observational studies have documented harbor seal responses to approaching vessels in non-glacial [21–24] and glacial habitat [1–4], to our knowledge no study has utilized data from marked (e.g., radio-tagged) seals to assess factors, including vessels, that affect haul-out behavior of individual harbor seals in glacial habitat. In observational studies, the severity of a vessel disturbance and the potential for lasting effects from that disturbance have been gauged by assessing how quickly the numbers of seals hauled out return to pre-disturbance levels [22, 24]. If seals disturbed by vessels leave the area shortly after a disturbance event, but an equal or greater number of different seals haul out at the same site shortly thereafter, the conclusion of little effect would be based on misinformation. Only by documenting the behavior of individually marked or radio-tagged seals over an extended time period can we assess whether vessel disturbance results in changes in haul-out behavior that reduce time spent resting, which could increase energetic expenditure [26] and potentially decrease fitness.

Factors other than vessels can also influence seals. Numerous studies have assessed haul-out behavior of unmarked harbor seals in terrestrial habitat as a function of covariates such as date, time of day, tide, and weather [27–31]; however, comparatively few studies have been conducted on seals in glacial habitat. One study of harbor seals in a glacial system (Glacier Bay National Park) revealed that seals utilizing tidewater-glacier habitat (i.e., glacial seals) had markedly different activity budgets and foraging strategies. Glacial seals traveled farther to forage, yet generally had better-quality diets compared to seals using terrestrial habitat in the Park, and glacial seals spent more time resting [32]. Similar body condition of seals in both habitats suggested that glacial seals balanced a trade-off of constantly available substrate (icebergs) to haul out on to rest after returning from foraging trips, with an apparent need to travel farther to find prey than did their terrestrial conspecifics [32]. Vessel traffic is restricted in some parts of Glacier Bay [3], including the primary glacial inlet used by seals [6, 33], which may have allowed those seals to spend more time resting on icebergs and may have influenced their movements and foraging strategies in that National Park.

We investigated behavior of harbor seals in glacial habitat within Tracy Arm Ford’s Terror (TAFT) Wilderness Area in southeast Alaska, where vessel traffic is unrestricted and growing in numbers each year. This area is frequented by cruise ships and day-trip or multi-day tourism vessels on their way to or from Juneau, Alaska, or other nearby ports. The primary objectives of this study were to: 1) assess the probability that a seal was hauled out relative to predictor variables including seal gender, environmental and temporal factors, and the presence of vessels; 2) evaluate those predictor variables relative to their influence on the duration, start and end of haul-out bouts; and 3) determine optimal times for vessel visitation to minimize disturbance to harbor seals.

## Methods

### Study area and vessel traffic

**Fig 1:**
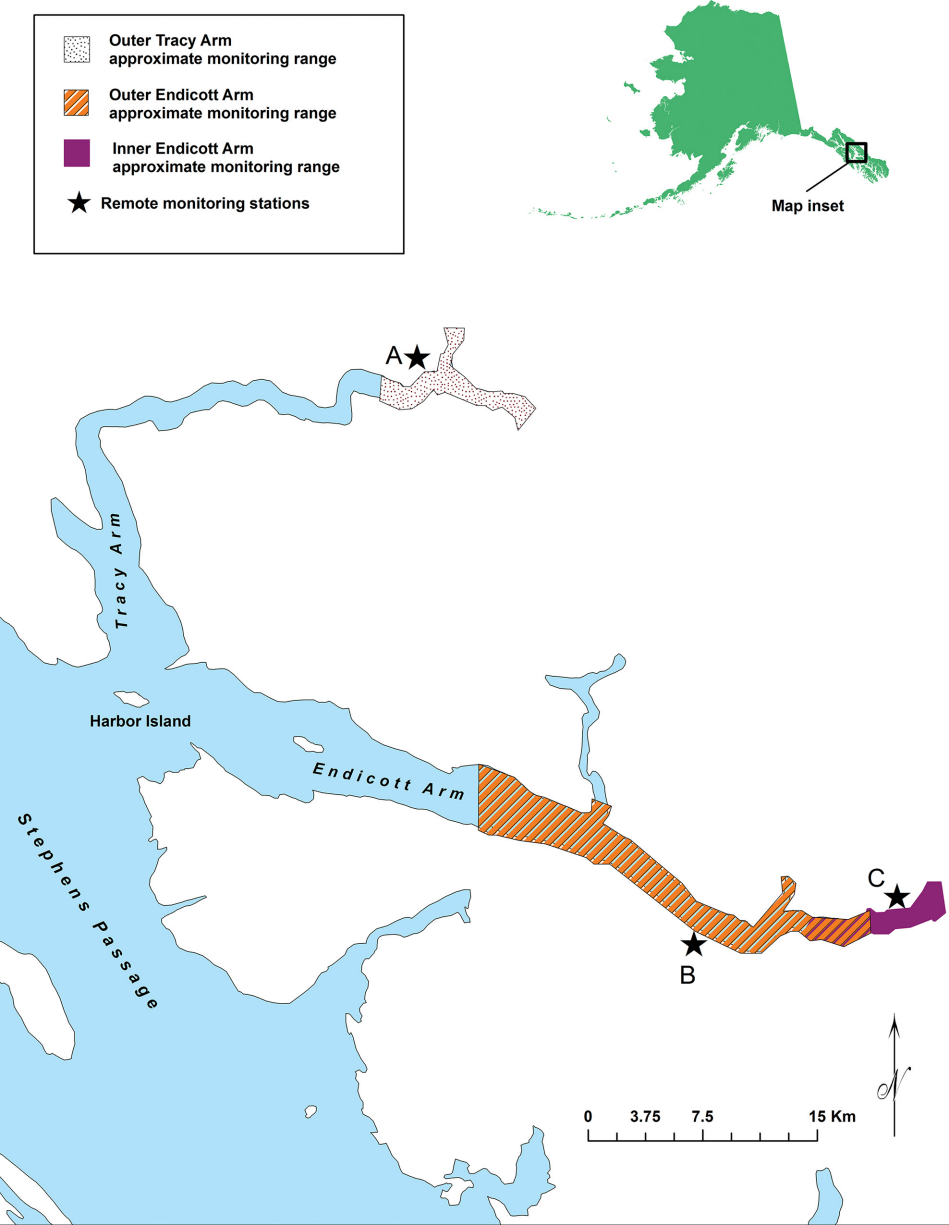
Location of the study area and three remote monitoring sites (stars). Each station was equipped with time-lapse cameras and VHF-telemetry data loggers. Shaded areas indicate the reception ranges at which the data loggers could detect a signal from a transmitting VHF radio tag.

Tracy Arm Ford’s Terror (TAFT) Wilderness area is located in southeast Alaska (57.759270° N, 133.627150° W; Fig 1) approximately 82 km southeast of Juneau, Alaska. TAFT is comprised of two adjacent glacial inlets—Tracy Arm and Endicott Arm. A total of 177 harbor seals were captured in Endicott Arm between 27 April and 10 May 2008–2010. Thick ice in Tracy Arm in 2008 and 2009 prevented access to seals during those capture efforts. Although ice conditions were more suitable for captures in Tracy Arm in 2010, for inter-annual consistency, we conducted captures only in Endicott in all three years of the study.

Vessel traffic is not officially regulated in this wilderness area. Nonetheless, an agreement (Wilderness Best Management Practices [WBMP] for Tracy Arm—Ford’s Terror Wilderness) exists between the U.S. Forest Service (USFS), Tongass National Forest, and tourism vessel operators using the area. The intention of the agreement is to maintain the wilderness character (e.g., preserving clean air and quiet), protect wildlife (e.g., vessels are advised to maintain >91m distance from seals on icebergs and minimize speed to reduce wakes), and preserve solitude by coordinating scheduling of tourism vessels to minimize simultaneous presence in the same Arm. The agreement—amended annually as necessary after discussions between vessel operators, tourism companies, and the USFS—involves voluntary compliance and requests that vessels with >250 passengers visit Tracy Arm while vessels with <250 passengers visit Endicott Arm. When unavoidable conflicts in scheduling would result in too many large vessels in Tracy Arm or when fog or ice conditions prevent safe navigation in Tracy Arm, larger vessels visit Endicott Arm. While all passengers generally remain aboard cruise ships and larger vessels, some of the large vessels with <250 passengers deploy kayaks or small skiffs which allow passengers to travel among the icebergs and get closer to the glacier face. Harbor seals are present in both Arms during pupping, breeding, and molt, and generally tend to haul out on icebergs that are densely packed and/or closer to the glacier face (Blundell pers. obs.).

### Ethics Statement and Animal Handling

The U.S. Forest Service, Tongass Region, issued permits for land-based monitoring stations in a designated wilderness area in all years of the study and for a field camp in 2009. The field studies did not involve endangered or threatened species.

Seals were captured using monofilament gillnets (30-cm stretch mesh, 7.5m deep and 60m long) deployed from inflatable skiffs (<6m). As seals became tangled in the capture net, they were pulled into a skiff, disentangled, and contained in hoop nets [34] for transport to a chartered vessel for processing and sampling. During processing, seals were weighed (to the nearest 0.1kg) and curvilinear body length and axial girth (to the nearest cm) were measured. Age of seals was estimated morphometrically [35]. Seals were physically restrained to draw blood from the caudal epidural vein to assess health, condition, and diet for several companion studies. Following the initial blood draw, seals were sedated with 0.25 mg kg^-1^ of diazepam administered intravenously to obtain blubber, skin, hair, and whisker samples for companion studies, and to attach radio tags.

A subset of captured seals (53 females and 54 males) were equipped with VHF transmitters; 99 received head-mounted VHF transmitters (MM 340B, 92g, Advanced Telemetry Systems (ATS), Isanti, MN, USA). In addition to headmounts, for a companion study, 49 seals received back-mounted VHFs (ATS MM 230B) included with other instrumentation in a flotation package. An additional 8 seals had a VHF backpack with no head-mount. All instruments were attached to the pelage with fast-setting epoxy and were shed by the seal 2.5–4 months later, during the annual molt.

The animal handling methods described herein were reviewed and approved by the Alaska Department of Fish and Game (ADFG) Institutional Animal Care and Use Committee (IACUC) protocol #07–16. Additionally, all animal handling, sampling procedures, tagging methods, and tag configurations were in strict accordance with research methods approved by a National Marine Fisheries Service (NMFS) Research Permit #358–1787. During the application process for receiving a USFS permit to conduct land-based activities in a wilderness area, the objectives of the study were reviewed and endorsed by Tongass Forest personnel of the USFS. The study design, describing animal handling methods, was provided to the USFS as a courtesy; however, the USFS has no jurisdiction over activities that occur on marine waters and does not have the required expertise to evaluate animal-handling methods for marine mammals.

### Remote monitoring of telemetry and vessel traffic

Three land-based monitoring stations were established in TAFT to remotely monitor seal presence via VHF telemetry and vessel traffic via time-lapse photography. Two monitoring stations were set up in Endicott Arm; one was located on a 200m cliff top, approximately 2.15km from the glacier face (inner Endicott), and a 2nd station (outer Endicott) was located 17.5km from the glacier face (Fig 1). A third monitoring station was set up in Tracy Arm (outer Tracy) approximately 6.75km from the glacier face (Fig 1).

We documented presence/absence of radio-tagged seals using VHF receiver-data loggers (R4500S, Advanced Telemetry Systems, Isanti, MN, USA) that continuously scanned (10 seconds/frequency) for all VHF frequencies deployed on radio-tagged seals. Reception range of telemetry signals was determined by observing the recording of detection of a test transmitter by each R4500S as a skiff bearing the transmitter traveled away from each station. The observer at the monitoring station was in radio contact with the skiff operator. Once the signal was no longer detected by the data logger, the skiff backtracked until the signal was again received and a GPS track was recorded to mark the perimeter of the reception range. Signal reception distance ranged from 3.2 to 16.8km (average 7.4km; Fig 1), depending upon the site and local topography.

To ensure that we were collecting reliable data, rather than false positives from ambient electronic interference, several data veracity “filters” were incorporated. A reference transmitter, that transmitted a signal 24h/day, was permanently positioned near each monitoring station and the frequency was programmed into the data logger and configured to be logged on every scan cycle, verifying that the equipment was operational. Dummy frequencies (i.e., frequencies that were not known to be deployed in the area) were programmed in and scanned for. If dummy frequencies were recorded as present, any legitimate frequencies that were also recorded as present were evaluated to ensure they represented accurate data (i.e., were also recorded before and/or after logging dummy frequencies).

Vessel presence was documented using Nikon Coolpix 8700 cameras with Lexar 8GB or 32GB CF camera cards, and a Digisnap 2000 electronic shutter release (Harbortronics, Fort Collins, CO) that programmed cameras to take photos at specific time intervals. The camera system was placed in a weather-tight housing along with a desiccant pack to control moisture. The camera configurations that we used were assembled by the Alaska Fisheries Development Foundation (Anchorage, AK). We programmed cameras to take photos every 15 minutes in 2008, and in 2009–10 cameras took photos every 5–8 minutes during daylight hours (05:00–22:00). Cameras were positioned for viewing down the length of the inlets rather than across, to increase potential for detection of vessels. Camera and telemetry equipment at each monitoring station were powered with an 80-watt solar panel and voltage regulator, and two sealed, 6-volt 240ah marine-grade batteries (PVX-2240L AGM, SunXtender, West Covina, CA) wired in series.

The site established at outer Endicott (Fig 1) had a longer viewing range down the inlet than did the camera established at inner Endicott. Ice coverage was generally low in outer Endicott and vessels passing by that camera were invariably bound for inner Endicott, venturing as far as the ice would allow them to proceed. High ice coverage that might impede vessel passage generally did not occur west of the area where the telemetry-reception range overlapped for both Endicott sites (Fig 1). Large-sized vessels (≥ 50m) often stopped at the outer periphery of thick ice (i.e., potentially before they reached the viewing range of the inner Endicott camera), thus the main function of the camera at outer Endicott was to document the passage of vessels that might not be detected by the inner camera.

### Data Analysis

#### Telemetry

We programmatically sorted and filtered data from remote monitoring sites to ensure data quality. Good data were sorted into two files: a summary file with the number of locations/seal/day, pulse rate, and site of those locations; and an individual location file with time, place, and the pulse rate each time a specific frequency was recorded as “good” data. Bad data (e.g., incorrect pulse rates) were sorted into various files that identified error type and data-logging location. Additional telemetry locations were obtained during vessel-based and aerial telemetry flights, but the majority of presence/absence data were obtained from the data-loggers, scanning for radio-tagged seals 24h/day.

For the first year of remotely-logged data, we examined >6000 records of “error” data to evaluate the validity of seal frequencies that were logged as present within 30 minutes of logging a “dummy” (non-existent) frequency and cross-referenced those error data with ~52,000 records of error-free, logged data. In all cases, the seal frequencies logged had correct pulse rates and no other error component other than temporal proximity to logging of a dummy frequency. In all but two cases, error-free detections were logged for that seal immediately before and after the dummy-associated detection; the two exceptions did not have locations logged for that seal prior to the dummy-associated detection, but had multiple error-free detections logged immediately thereafter. Because the detailed evaluation of one season of data did not reveal any apparent problems with data logged in association with the detection of dummy frequencies, we concluded that deleting otherwise error-free seal data associated with logging of dummy frequencies was not warranted. Accordingly, we did not manually inspect or reject data logged in the two subsequent years that were logged within 30 minutes of logging of dummy frequencies.

Some seals were double-tagged with head-mounted VHFs and (for a companion study) backpacks that contained a Time Depth Recorder (TDR) and VHF transmitter within a float pack. When seals were hauled out, a telemetry signal from the backpack was detectable; however, transmitters shed by the seal and floating in the water were also detectable. To avoid quantifying a molted tag as seal behavior, data from recovered TDRs was inspected to determine when the last dive was taken. Any subsequent telemetry data past the date of the last dive were trimmed from the dataset.

#### Vessel photos

All photos obtained during time-lapsed photography were examined for vessel presence. When vessels were present, the location, vessel type, and time of entry and departure for each vessel were determined. Vessels were classified according to size as follows: 1) Cruise Ships (>90m in length); 2) Large (≥50m) including tourism vessels and privately owned motor vessels and sail boats; 3) Medium (≥4.5m <50m) privately owned motor vessels and sail boats; 4) Small (<4.5m) including skiffs and inflatables; and 5) kayaks. Kayaks were examined as a separate vessel class because previous research [22, 24] noted that seals may be more sensitive to their presence than to other vessel types.

To estimate camera detection rates for a subset of vessels, we compared camera “captures” of passing cruise ships and large vessels with data on vessels that were known to be present in the general area as a result of the Automatic Identification System (AIS) transponders aboard those vessels. AIS transponders automatically transmit the GPS coordinates and velocity of the vessel at regular intervals via a VHF radio, built into the transponder. All vessels >300 metric tons (i.e., cruise ships) and vessels licensed for international travel are required by law to have AIS transponders. Other vessels can voluntarily use AIS to allow vessel operators to see and be seen by other vessels with AIS to aid in collision avoidance and safer navigation, and to facilitate rapid location and rescue in the event of an emergency. Numerous tourism vessels and some private vessels entering TAFT were equipped with AIS.

During this study, the spatial coverage for documenting movements of AIS vessels did not extend to the head of either Tracy or Endicott Arm. Because glacier viewing is the primary activity for vessels in TAFT, we assumed that AIS-equipped vessels that entered either Arm would travel as close to the glacier as the ice conditions would permit. Our use of AIS data is only valuable as a means to assess whether time-lapse photos were adequately representing, or were underestimating vessel activity. These AIS data cannot be used to evaluate behavior of seals in the presence of vessels because AIS coverage in Tracy Arm did not overlap with the telemetry-reception range (i.e., the spatial limits of our seal behavior data). In Endicott, AIS and telemetry data only overlapped in the outer approximately one-third of the telemetry range for outer Endicott, in an area where seals seldom hauled out.

Photos were also used to evaluate weather conditions and ice coverage. Sky conditions were categorized into four groups (foggy, overcast, partly cloudy, and clear). We selected example photographs that were characteristic of each weather condition and compared the example photos to each time-lapse photograph evaluated in order to maintain consistency throughout the course of the subjective ratings. All photographic evaluations were conducted by the same person. Percent cloud cover was estimated in a similar way; photographic examples of cloud cover were selected that were representative of 0%, 5%, 10%, increasing by 10% increments thereafter up to 100%, those photos were compared to each photograph evaluated. Generally, foggy and overcast weather conditions were associated with a 100% cloud cover, and clear weather conditions were indicated by 0–10% cloud cover. Photos were also evaluated for evidence of precipitation (yes/no), where precipitation (rain) was perceived by the presence of droplets on the camera lens, observation of precipitation shafts beneath the clouds, or by conditions in which there was little contrast beneath the clouds due to flat lighting. Ice cover was evaluated by estimating percent ice cover in the photograph and designated in the following categories: Low < 33%, Medium = 33–66%, and High > 66%. Example photographs of ice categories were selected and compared to every photograph in each Arm for consistency.

#### Statistical Analyses

We analyzed four response variables associated with seal behavior in relation to seal characteristics, environmental variables, and the presence of vessels. The four responses were: 1) the probability that a seal was hauled out; 2) the mean duration of a haul-out bout; and conditions associated with either 3) the start; or 4) the end of a haul-out bout. For these responses, we only used data when the seal was considered to be present ‘in’ one of the monitored inlets.

Harbor seals in Alaska spend 40–80% of their time in the water [36, 37] and VHF radio-transmitters are only detected when the transmitter antenna is above the surface of the saltwater. A seal may be present in the area and surfacing for brief intervals between foraging or traveling dives [38] and therefore not likely to be detected during scans of radio frequencies. When assessing factors that influence haul-out behavior, excluding seals that were present but not detected because they were intermittently diving could potentially underrepresent actual behavior. Our analyses therefore used a conservative approach to define seal presence by delineating a time frame in which a seal was considered to be within either of the monitored glacial inlets. ARGOS satellite telemetry for harbor seals previously tagged in TAFT for another study (ADF&G unpublished) revealed that some seals left glacial habitat for periods of time ranging from days to weeks before returning, while others appeared to forage among the icebergs or ventured away from the proximity of the glacier for a few hours at a time. Our unpublished satellite-telemetry data identified a foraging location near Harbor Island (Fig 1), approximately 5–6 hours travel time (based upon the reported swim speed of harbor seals [39, 40]) from the telemetry detection-range within glacial habitat in TAFT (Fig 1).

Therefore, in our analyses, if no signal from a seal was detected for 5 hours, the seal was considered to be out of the inlet. We reasoned that 5 hours was a realistic amount of time in which seals might be present but not hauled out if they were foraging among icebergs, or were within the detection range of our telemetry equipment but not hauled out as a result of returning from foraging at Harbor Island. For seals that had been out of the area, we defined the seal to be ‘in’ the inlet as starting 5h before the first detection after an ‘out’ interval.

All haul-out behavior was presumed to be on ice rather than land, as there was only one small, land-based haul-out site within range of the telemetry stations that was frequently submerged at higher tides and was rarely used by seals. To organize the data, the haul-out status of each seal was determined when a scanning telemetry receiver (at any of three locations; Fig 1) detected a signal from the seal. If >1 signal was detected ≤ 18 minutes apart, we considered the seal to be hauled out; signals at longer intervals were considered to be from a swimming or diving seal. The cutoff of 18 minutes was determined based on how long it took the receiver to scan through the frequencies of all of the seals available that year. So long as signals continued to be received at intervals <18 minutes, a seal was considered to be consistently hauled out; an interval >18 minutes terminated what we considered to be a haul-out bout. For each haul-out bout, we then defined the start or end of the bout to be 9 minutes before the first received signal of the bout, or 9 minutes after the last signal of the bout; 9 min. being half of our cutoff interval. The duration of the haul-out bout was the difference between the start and end of each bout. To estimate the probability that a seal was hauled out, we summarized status and predictor variables every 15 minutes throughout the day. If a 15 minute point fell during a haul-out bout, the status for that point was ‘hauled out’; otherwise the status for that point was ‘not hauled out’.

To investigate factors associated with the end of haul-out bouts, we defined the end point as the last of the 15 min. interval points from each haul-out bout. For comparison, we examined factors associated with continuation of a haul-out bout by selecting the 15 min. point from the middle of each haul-out bout; if the number of points in a haul-out bout was odd, the middle point was used, but if the number of points was even, we used the point from rounding down from *n*/2, where *n* is the number of points for the bout. For the haul-out end analyses, we excluded bouts with ≤5 points under the assumption that the predictors would be less likely to differ between the middle and the end of a bout when there was only 30 minutes between them.

#### Presence in an Inlet

To estimate the probability that a seal was in one of the inlets in relation to the predictor variables, we used mixed-effect generalized linear models [41]. Because the response variable was binary (i.e., in an inlet vs. out of the inlets), we used a logit link and a binomial error structure in these models. For predictor variables in these analyses, we used year, season (1 = before 1 June, 2 = 1–15 June, 3 = 16–30 June, 4 = 1–15 July, 5 = after 15 July), and sex. Seal age (e.g., young of the year, juvenile, subadult, or adult) was not used as a predictor because, while other age classes were well represented (≥29/age class), there were only 5 adults in the sample. We also included interaction terms year*season and season*sex. Other variables that changed on a more rapid time scale (e.g., hour of the day, ice cover, presence of vessels), were not appropriate predictors for the probability of being in an inlet, which changed on a longer time scale.

Because observations for an individual seal were not independent, ‘seal’ was included as a random effect, as was the interaction of seal and predictor variables other than ‘sex’ or year, which cannot interact with seal (i.e., an individual seal has only one sex and was monitored in only one year). We selected models by fitting the most complex model for a given dataset, then deleting unimportant variables based on Wald F statistics; degrees of freedom were determined using Satterthwaite’s approximation [41].

#### Haul-out Probability

We analyzed the probability that a seal was hauled out using models and procedures similar to those used for estimating the probability that a seal was in an inlet. For these models, we used the predictors location (inner Endicott Arm, outer Endicott Arm, outer Tracy Arm), season, sex, hour of the day, tidal flow (falling, rising, slack), ice cover (low, medium, high), precipitation (no, yes), sky condition (clear, partly cloudy, foggy, overcast), cloud cover (%), vessels (continuous: the number of vessels present for each of 5 types of vessels or the total number of vessels), and vessels (binary: absence/presence if ≥1 vessel of a type was present). Some of these variables are strongly related. Thus, to facilitate model fitting, environmental predictors (i.e., ice, precipitation, sky condition, and cloud cover) were included in models singly, rather than simultaneously.

We also included interaction terms year*location, season*location, season*sex, and season*hour. Vessel variables were included in models separately (i.e., only 1 type of vessel in a single model). Models with only time (i.e., year, season, and hour), location, sex, and tidal flow, and their interactions, used all data. Models including ice, precipitation, sky condition, cloud cover, and vessels, used data from 05:00–20:59, because these variables were based on photographs, which required enough light to determine the values of the variables. Further, models with vessel variables omitted data from 2008; photographs taken in 2008 were 15 minutes apart, making vessel counts unreliable (e.g., vessels could enter or leave without being detected). In 2009 and 2010, photographs were taken every 5–8 minutes, making the vessel data more reliable.

#### Haul-out Duration

We modeled mean duration of haul-out bouts as a function of covariates using mixed-effects linear models [41]. Because haul-out durations were highly skewed (many short bouts and few long bouts), we analyzed the natural logarithm of duration; results are transformed back to the original scale for presentation. We used predictor variables year, season, location, sex, hour at the start of the haulout (hr_start), hour at the end of the haulout (hr_end), tidal flow, ice cover, sky condition, precipitation, cloud cover (%), vessels (continuous), and vessels (binary). We also included 2-way interactions of season, location, sex, hr_start, and hr_end. Hr_start and hr_end were not used in the same models. We used the same subsets of the data for analyses of variables collected only during daylight and for the vessel data as described for the analyses of haul-out probability. We again used Wald F statistics and Satterthwaite’s degrees of freedom for selecting models.

#### Haul-out Start and End

For both start (i.e., the first observation in a haul-out bout) and end (i.e., the last observation in a haul-out bout)–‘starts’ and ‘ends’ were analyzed separately—we modeled the probability that the observation was a start or an end observation vs. a mid-bout observation using mixed-effects generalized linear models (logit link, binomial error; [41]). To predict the probability of a bout start or end, we used predictor variables hour of the day, tide flow, ice cover, precipitation, sky condition, and the presence of vessels (binary). Precipitation, sky condition, and cloud cover are strongly related and were included in models singly, rather than simultaneously.

We also included the interactions sex*hour, season*hour, and sex*vessels. Because of the paired nature of the data (1 start, 1 middle and 1 end observations from each haul-out bout), some predictors are balanced and are not useful predictors of end vs. middle observations (e.g., year, season, location, sex). We used the same data subsets and model selection as for the analyses of haul-out probabilities and haul-out durations.

#### Covariate Effects Relative to Time of Day

To help interpret the relationships between seal behavior and environmental covariates (e.g., sky condition, precipitation, cloud cover, tidal stage, ice conditions, or vessel presence), we modeled the relationships between the covariates and time of day using mixed-effects generalized linear models (logit link, binomial error; [41]). Specifically for each covariate, we estimated the probability that observations in each covariate category occurred in one of four time blocks during the daylight hours monitored by the cameras: early morning (0500–0859); late morning (0900–1259); afternoon (1300–1659); and evening (1700–2059).

## Results

### Telemetry

Three seals were never located following radio tagging. An average of 1,633.9 total locations (SE = 110.6, minimum = 0, maximum = 6011) were obtained per seal. Elapsed days of radio-tracking seals averaged 61 days (SE = 2.6, min = 1, max = 112); there was no difference in numbers of days tracked for males vs. females (*t* = 0.798; *p* = 0.4 two-tailed, two-sample t-test assuming unequal variance). On average, seals were tracked through the end of July. Telemetry-monitoring stations were functional for a total of 894 days during the study (Table 1); 273 total days in 2008 (average 91days/station), 292 days in 2009 (average 97.3 days/station), and 329 days in 2010 (average 109.7days/station). The majority of locations were in inner Endicott, where seals were tagged. In 2008, 92.9% of locations were in inner Endicott with 0.6% in outer Endicott and 6.5% in Tracy Arm. In 2009, 89.3% of locations occurred in inner Endicott, 4.8% in outer Endicott, and 5.9% in Tracy, and in 2010, 63.5% of locations were in inner Endicott, 29.5% in outer Endicott and 7% in Tracy.

**Table 1:** Dates and locations of telemetry coverage.

Location	Year	Start Date	End Date	Total Days of Coverage	Dates w/o Coverage
**Inner Endicott**	2008	9-May	29-Aug	108	5/10, 5/12-5/15
	2009	12-May	26-Aug	107	none
	2010	13-May	2-Sep	104	8/8, 8/10-8/11, 8/13-8/15, 8/21, 8/25, 8/29
**Outer Endicott**	2008	20-May	7-Aug	59	6/7,6/16-6/17, 6/22, 6/24, 7/19, 7/22-7/23, 7/25-8/6
	2009	13-May	26-Aug	95	6/15-6/26, 6/28
	2010	12-May	1-Sep	113	none
**Outer Tracy**	2008	15-May	28-Aug	106	none
	2009	15-May	25-Aug	90	6/14-6/23, 8/15-8/17
	2010	12-May	31-Aug	112	none

Data were obtained via remote monitoring with R4500S ATS receiver/data loggers.

### Cameras

**Fig 2:**
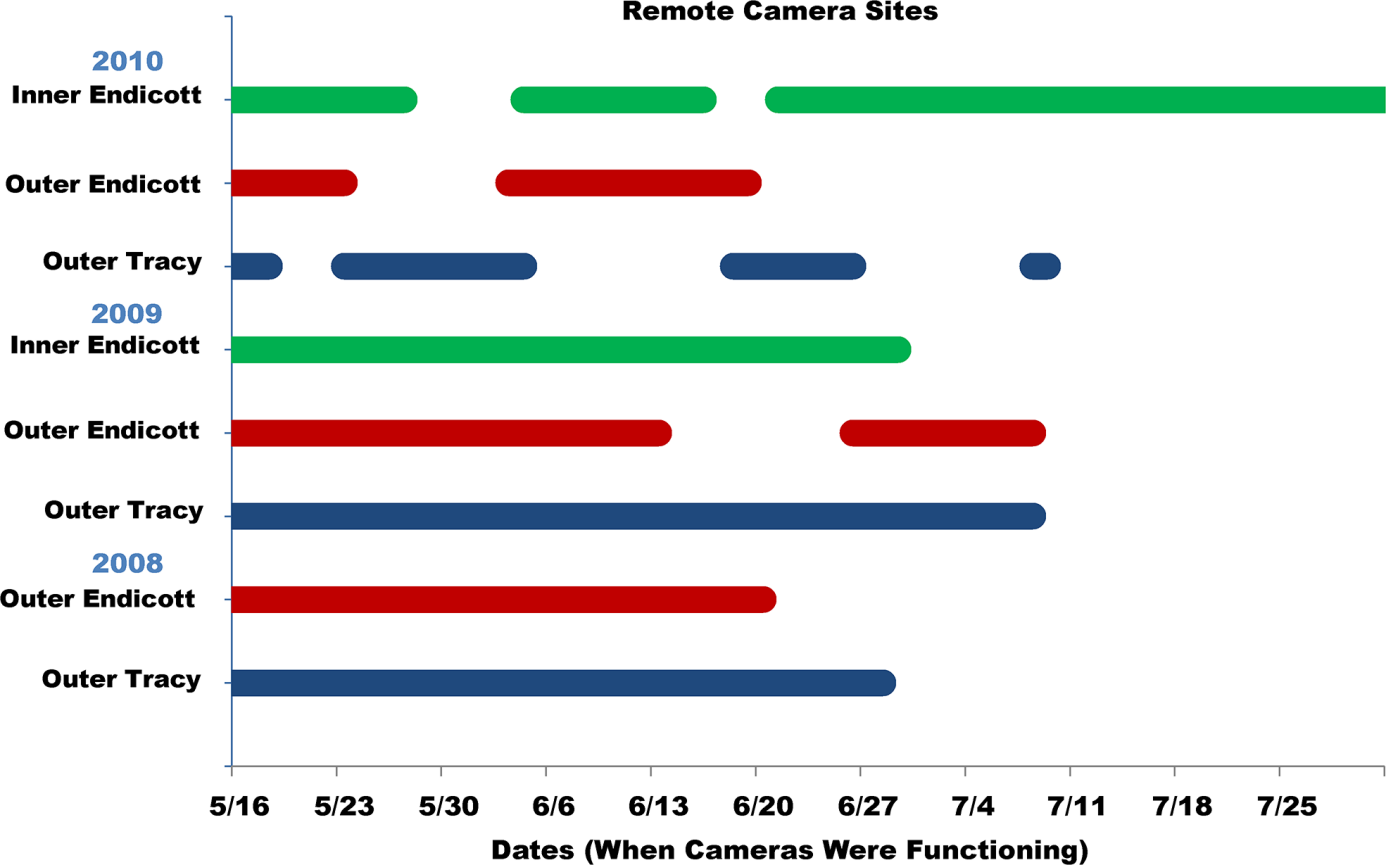
Dates of operation for time-lapse cameras. Cameras were used to detect vessel presence and assess environmental variables such as weather conditions and ice coverage. Horizontal bars indicate the range of dates (color-coded by camera site) that each time-lapse camera was functional at each remote monitoring station throughout the years of the study.

Cameras were functional for a total of 511 camera-days (Fig 2) during the study. A subset of 427 camera-days in 2009–10 were used in the analyses of vessel effects on seal behavior: 256 camera-days in 2009 (47 in inner Endicott, 44 in outer Endicott, and 165 in Tracy Arm) and 171 camera-days in 2010 (96 days in inner Endicott, 47 in outer Endicott, and 28 days in Tracy Arm). Cameras successfully detected AIS-equipped vessels entering and/or exiting the study areas an average of 44.2% of the time for both years, all vessel types, and all cameras combined (Table 2). AIS-vessel detection rates were higher in Endicott than in Tracy Arm and higher in both Arms in 2009 than in 2010. A higher proportion of AIS-equipped vessels entering Endicott were detected by the outer camera than the inner camera in 2009, while the opposite pattern occurred in 2010. Two factors likely affected these results; larger vessels that deploy smaller vessels to travel farther into the ice and those more vulnerable to damage from icebergs often stop at the edge of thick ice and may not venture far enough into the inlet to be detected with the inner Endicott camera. Additionally, we had greater camera coverage in inner Endicott in 2010 and problems with the outer Endicott camera that year when the camera at that site was repeatedly disturbed by bears. On several of those occasions the camera remained functional but the angle of view of outer Endicott was altered, changing from a long-range view down the arm in the direction of the mouth of the inlet to a short-range view across the arm, substantially reducing the potential for detecting passing vessels.

**Table 2:** Comparison of AIS data for vessel entry vs. camera detections of AIS-equipped vessels.

Location	Vessel Type	AIS Total Known Entries	# Detected Any Camera	% Detected Any	% Detected Outer	% Detected Inner
**Endicott**		**AIS # 2009**				
	Cruise Ship	37	23	62.2%	56.8%	8.1%
	Large	19	13	68.4%	57.9%	26.3%
	**Total**	**56**	**36**	**64.3%**	**57.1%**	**14.3%**
		**AIS # 2010**				
	Cruise ship	15	4	26.7%	6.7%	20.0%
	Large	48	28	58.3%	22.9%	45.8%
	**Total**	**63**	**32**	**50.8%**	**19.0%**	**39.7%**
**Tracy**		**AIS # 2009**				
	Cruise ship	57	34	59.6%	59.6%	NA
	Large	51	25	49.0%	49.0%	NA
	**Total**	**108**	**59**	**54.6%**	**54.6%**	
		**AIS # 2010**				
	Cruise ship	74	11	14.9%	14.9%	NA
	Large	90	13	14.4%	14.4%	NA
	**Total**	**164**	**24**	**14.6%**	**14.6%**	

Comparison of numbers and percentage of AIS-equipped vessels known to be entering Endicott and Tracy Arms, according to AIS data, compared to numbers detected at each camera site in 2009 and 2010 in each Arm.

Telemetry detections with missing data (i.e., no vessel data due to nonfunctional cameras) were dropped from the analyses; thus inference about vessel effects on seal behavior depended solely on observations when there were vessel data. This is a separate issue from undetected vessels (i.e., those not detected by the cameras). Such observations (i.e., false negatives) can affect the estimates of vessel effects, but likely only our ability to detect the effect. That is, undetected vessels would make it more difficult for us to detect a true effect of vessels on seals, but it would not introduce a vessel effect that didn’t actually exist. If, indeed, all AIS-equipped vessels traveled to well within the telemetry coverage, our AIS comparison indicates that cameras underestimated the presence of larger vessels. Smaller vessels were more difficult to detect among the icebergs and most moved faster, further reducing the potential to be photographed if they moved past cameras during the lapse between photos. Consequently, small vessels were at least equally as underestimated as large vessels, but likely more so; thus, our estimates of vessel effects on seals are conservative.

### Ice Availability and Vessel Traffic

**Fig 3:**
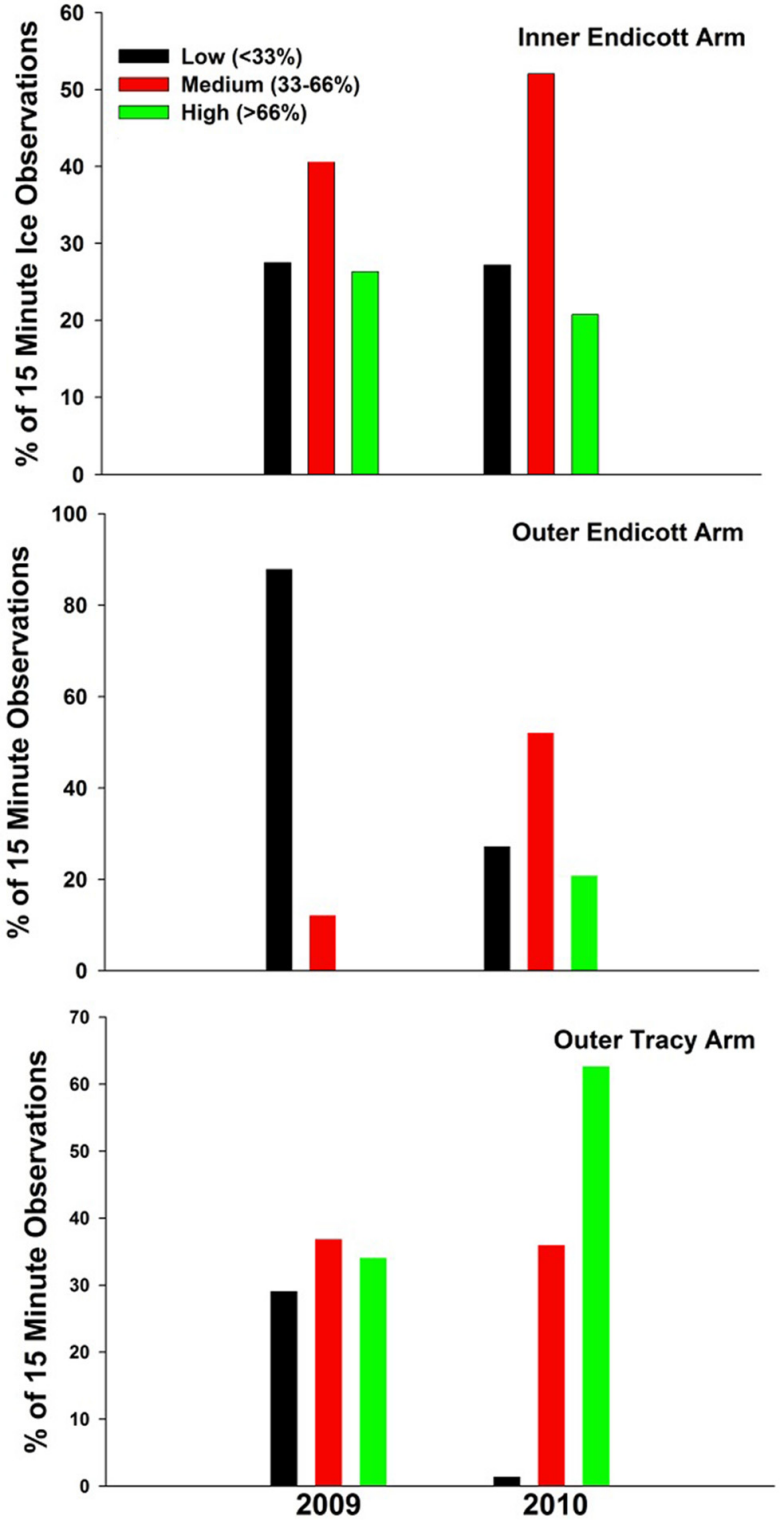
Percent ice coverage. The percent of all 15 minute observation periods in which three categories of ice coverage were observed in 2009 and 2010 for all three remote monitoring sites in the study area. The percentage of the area covered by icebergs within each time-lapse photograph was estimated and categorized in thirds, as low (<33% ice coverage), medium (33–66%), and high ice coverage (>66%).

**Fig 4:**
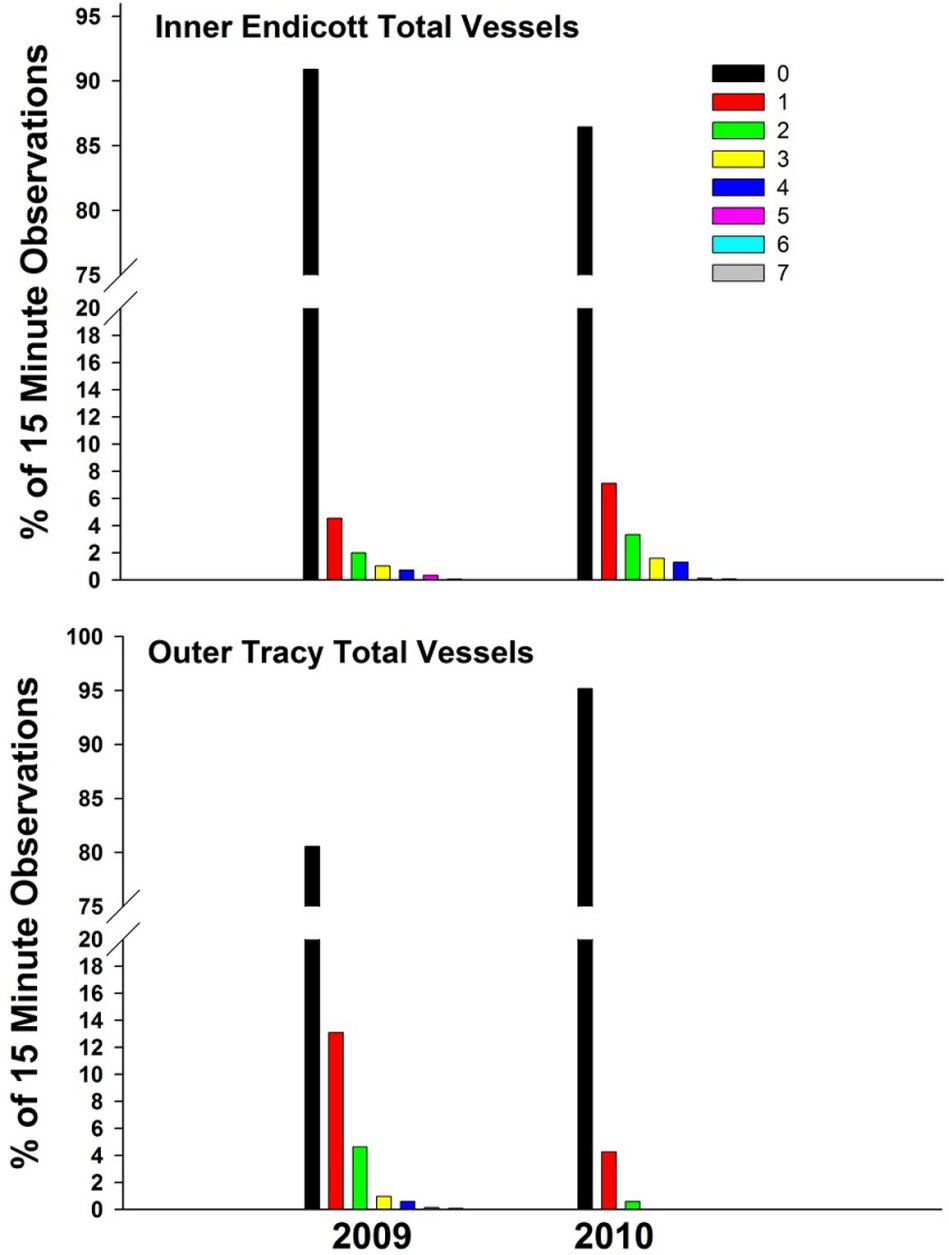
Percentage of observations when vessels were observed in the study area. Percent of 15 minute observation periods in which zero, one, or more vessels (all vessel-types combined) were present in inner Endicott Arm and in Tracy Arm in 2009 and 2010. Outer Endicott is omitted because vessels generally moved through the area to reach the inner Arm.

**Fig 5:**
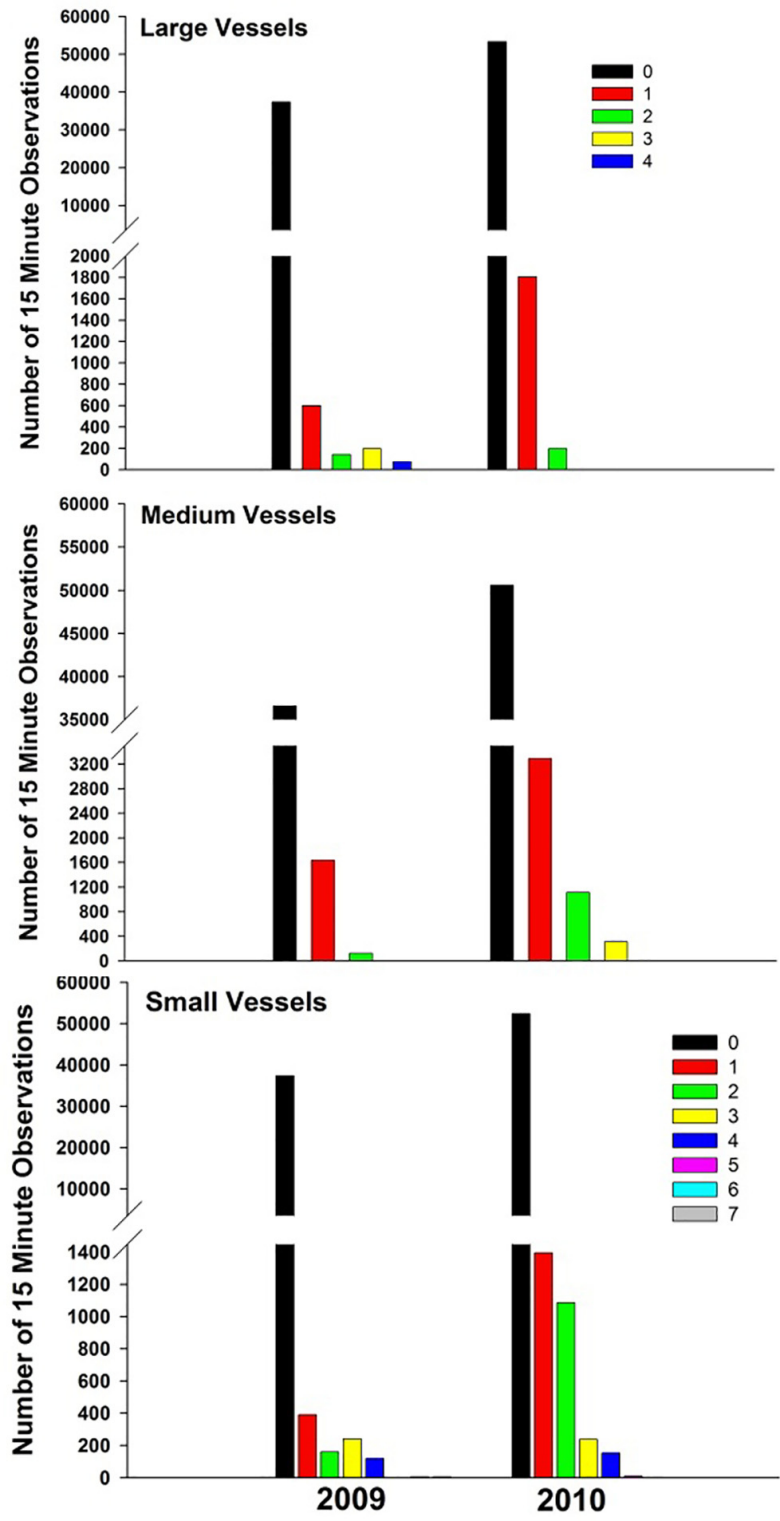
Extent of time that vessels were observed in inner Endicott Arm. Number of 15 minute observation periods in inner Endicott Arm when zero, one, or more vessels were present for the following size categories: Large (>50m; tourism vessels, private vessels, and sailboats), Medium (≥4.5m <50m; private vessels, and sailboats), and Small (<4.5m; skiffs and inflatables). Only inner Endicott is shown because the majority of seal telemetry-detections occurred there.

To better understand and compare the extent of ice and known vessel presence in both Arms and both years in light of unequal sampling effort due to intermittent camera function, we evaluated the percentage of 15 minute observations for which ice conditions (Fig 3) and vessels (Fig 4) were observed. An examination of the numbers of vessels of each type observed in those 15-minute time periods provided a means of visualizing the magnitude and duration of vessel traffic experienced by seals (Fig 5).

Ice availability varied among years and sites (Fig 3). The percent of 15 minute observations of low ice availability were identical between years in inner Endicott, with small differences in observations of medium (increased by 6%) and high ice coverages (decreased by 5%) for 2010 compared to 2009. Ice availability in outer Endicott was characterized by a predominance of low ice, and no observations of high ice availability in 2009. In contrast, in 2010 there was considerably more ice present; >1/2 of the observations were of medium levels of ice and 21% of observations were high ice (Fig 3) in outer Endicott. Tracy Arm had roughly similar percentages of low, medium, and high ice concentrations in 2009, however in 2010, 63% of observations were of high ice, 36% of medium ice and only 1% of the observations noted low ice concentrations (Fig 3).

Table 3 and Fig 4 document the numbers of vessels present in Endicott and Tracy Arms during 2009 and 2010 and the extent of time that they were there, respectively. In inner Endicott in 2009, in 91% of 15 minute observation periods, the camera did not detect any vessels, whereas in 2010, 86% of observation periods detected no vessels (Fig 4). For all vessel-types combined, a higher percentage of observations occurred where ≥1 vessels were detected by the camera in inner Endicott in 2010, compared to 2009 (Fig 4; Table 3). In Tracy Arm, the opposite pattern was observed, with fewer observations of ≥1 vessels in 2010 than in 2009 (Fig 4). The increase in vessel presence in 2010 was most notable for small, medium, and large vessels in inner Endicott (Fig 5; Table 3).

**Table 3:** Tally of numbers of observations of vessels captured with cameras by vessel category, inlet, and year.

	**Large**						**Medium**				
**Endicott**	**0**	**1**	**2**	**3**	**4**	**≥1**	**0**	**1**	**2**	**3**	**≥1**
2009	37,334	601	143	197	74	1,015	36,587	1,638	124	0	1762
2010	53,293	1,804	197	0	0	2,001	50,577	3,289	1,115	313	4,717
**Tracy**											
2009	9,346	621	153	16	4	794	9,079	905	142	14	10,140
2010	2,908	28	5	0	0	33	2,863	74	4	0	2,941
	**Small**										
**Endicott**	**0**	**1**	**2**	**3**	**4**	**5**	**6**	**7**	**≥1**		
2009	37,362	391	160	240	120	0	6	6	923		
2010	52,410	1,396	1,087	239	153	9	0	0	2,884		
**Tracy**											
2009	9,968	57	66	43	6	0	0	0	172		
2010	2,933	8	0	0	0	0	0	0	8		
	**Cruise Ships**				Kayak*						
**Endicott**	**0**	**1**	**2**	**≥1**	**0**	**1**	**2**	**3**	**4**	**5**	≥1**
2009	37,821	528	0	528	38,054	18	167	16	11	6	295
2010	55,081	213	0	213	55,294	0	0	0	0	0	0
**Tracy**											
2009	9,812	315	13	328	10,139	1	0	0	0	0	1
2010	2,910	31	0	31	2,941	0	0	0	0	0	0

Camera detections of vessels entering the study area reported as numbers of 15 minute observations by numbers of each vessel type observed, camera site, and year. Endicott Arm data are from detections by the inner Endicott camera. For easier visualization of the magnitude of the total numbers of vessels present, the ≥1 column is the sum of all columns >0.

* Kayaks were almost never detected by our cameras, and are thus under-represented in our data.

**In 2009, kayak counts were made from a field camp; there were up to 14 kayaks present in Endicott Arm at one time. The total ≥1 for kayaks that year includes 77 additional observation periods with >5 kayaks that are not shown in this table.

The majority of radio-tagged seals were detected in inner Endicott and the increase in duration of vessel presence (Fig 4) and numbers of vessels in that Arm (Fig 5; Table 3) likely influenced the quality of the habitat in which seals were hauling out. The number of observations of one or two large vessels detected by cameras in Endicott increased threefold in 2010 compared to 2009, increasing to 2001 15-minute observation periods, or ~500 hours. Detection of ≥1 medium or small vessels generally increased by at least two to four times the level of the previous year (Fig 5), representing a total of approximately 1200 and 700 hours, respectively, in which ≥1 vessel in each category was present (Table 3).

### Presence in an Inlet

**Fig 6:**
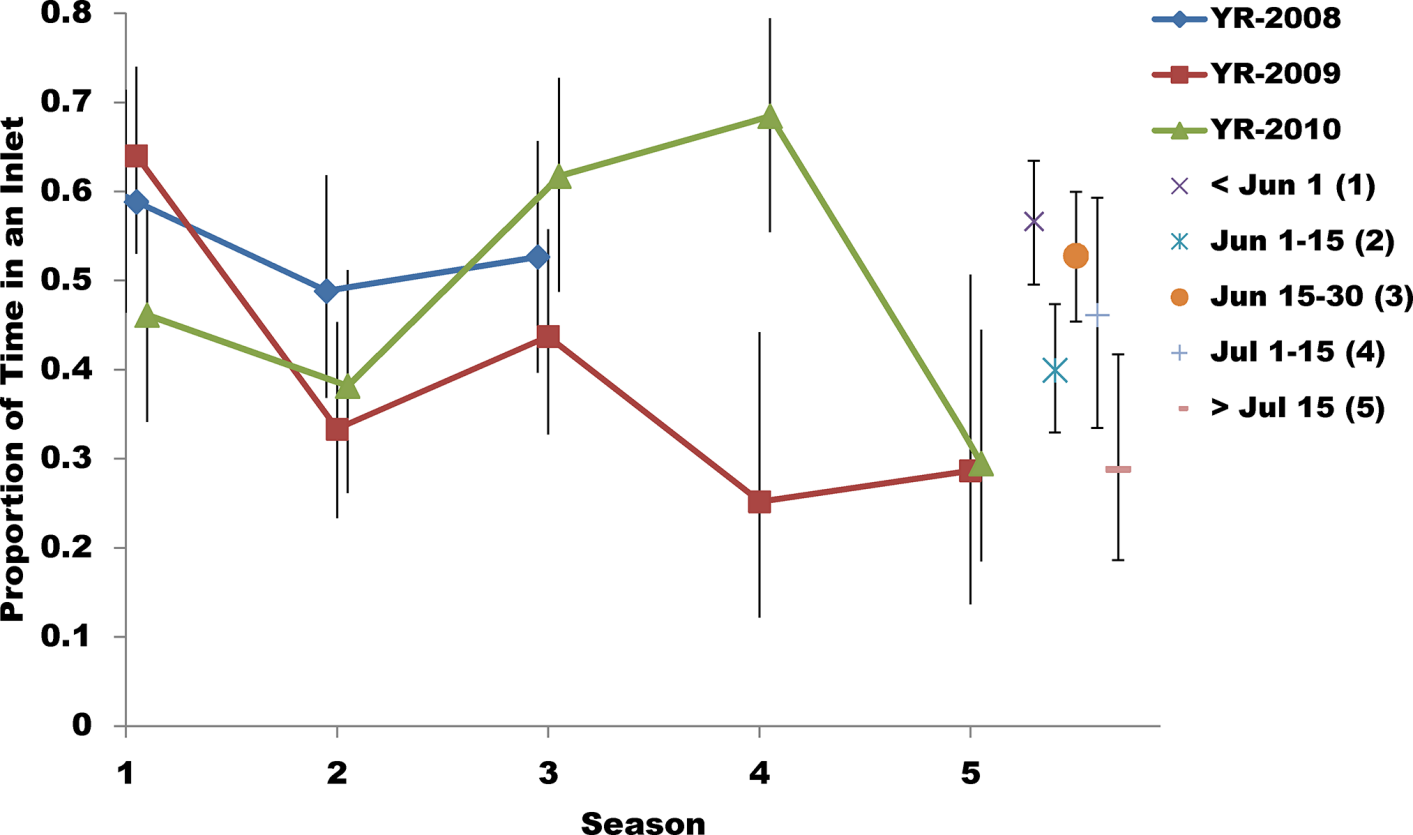
Proportion of time that radio-tagged harbor seals spent in glacial habitat. Proportion of time seals (n = 104; tagged in Endicott Arm) spent in Tracy or Endicott Arms by year and season. Vertical bars are 95% Confidence Intervals, by year, season, and location (on left), and (on right) by season (all years combined). Season numbers and their respective range of dates are in the legend.

The probability that a seal was in one of the glacial inlets (e.g., Tracy or Endicott Arm) was affected by season (p<0.001), with the seasonal pattern differing among years (year*season p<0.001; Fig 6). The average probability of a seal being in one of the inlets was not a function of year (p = 0.206), sex (p = 0.731), or season*sex (p = 0.724). The probability that an individual seal was in one of the inlets (i.e., in glacial habitat) varied from 0.075 to 1, with an average of 0.507.

### Haul-out Probability

Models that included weather covariates and ice availability in association with season*sex failed to converge; that interaction was omitted from models that included weather (precipitation, sky condition, ice, and cloud cover) and ice availability. Haul-out probability varied among years, locations, seasons, hour of the day, and with the presence of vessels; interactions of some of these variables also were important (Table 4). The main effects of sex and tidal flow were not useful predictors. Specifics on statistical analyses were described in the methods but are included in the footnotes of the tables for easy reference.

**Table 4:** Haul-out probability as a function of predictor variables.

Predictor	P-Value	Odds Ratio (95%CI)	Marginal Mean
**Year**	0.051		
2008			0.07 (0.04,0.11)
2009			0.08 (0.06,0.11)
2010			0.12 (0.09,0.17)
**Location**	<0.001		
Inner Endicott			0.25 (0.20,0.30)
Outer Endicott			0.014 (0.010,0.021)
Outer Tracy			0.16 (0.10,0.26)
Year*Location	<0.001		Fig 7
**Season**	<0.001		Fig 8
Season*Location	<0.001		
**Sex**	0.961		
Season*Sex	0.015		
**Hour of Day**	<0.001		Fig 8
Season*Hour	<0.001		Fig 8
**Tidal Flow**	0.282		
**Ice** ^**1**^	<0.001		
Low			0.09 (0.06,0.12)
Medium			0.12 (0.09,0.17)
High			0.14 (0.11,0.19)
**Precipitation** ^**1**^	<0.001		
No			0.12 (0.09,0.16)
Yes			0.09 (0.07,0.12)
**Sky Condition** ^**1**^	<0.001		
Clear			0.10 (0.08,0.14)
Partly Cloudy			0.12 (0.09,0.16)
Foggy			0.06 (0.04,0.08)
Overcast			0.11 (0.08,0.15)
**Cloud Cover** ^**1**^	0.480		
**Vessels** ^**2**^			
Cruise ship	0.003	0.56 (0.40,0.81)	
Large	<0.001	0.67 (0.57,0.78)	
Medium	0.019	0.71 (0.56,0.91)	
Small	0.030	0.72 (0.56,0.92)	
Kayak	0.414	0.60 (0.18,1.81)	
Vessels (All)	<0.001	0.67 (0.58,0.78)	

Odds ratios are the relative odds of a seal being hauled out for one value of the covariates vs. another value [47]. Marginal means (i.e., SAS least-squares means; [41]) are the estimated probability of a seal being hauled out for each level of the predictor variable, adjusted for other predictors in the model. Analyses with respect to ice, vessels, cloud cover, precip, and sky, were based on data from 2009–2010 and only for the daylight hours >0500 to <2200.

^1^Models containing these predictors omitted the season*sex interaction to facilitate convergence; these factors (i.e., ice, precipitation, sky condition, and cloud cover) were included in models singly, rather than simultaneously.

^2^Results for binary vessel predictors (i.e., absence/presence); results for the binary vessel variables are easier to interpret, however models using continuous vessel predictors showed similar patterns.

**Fig 7:**
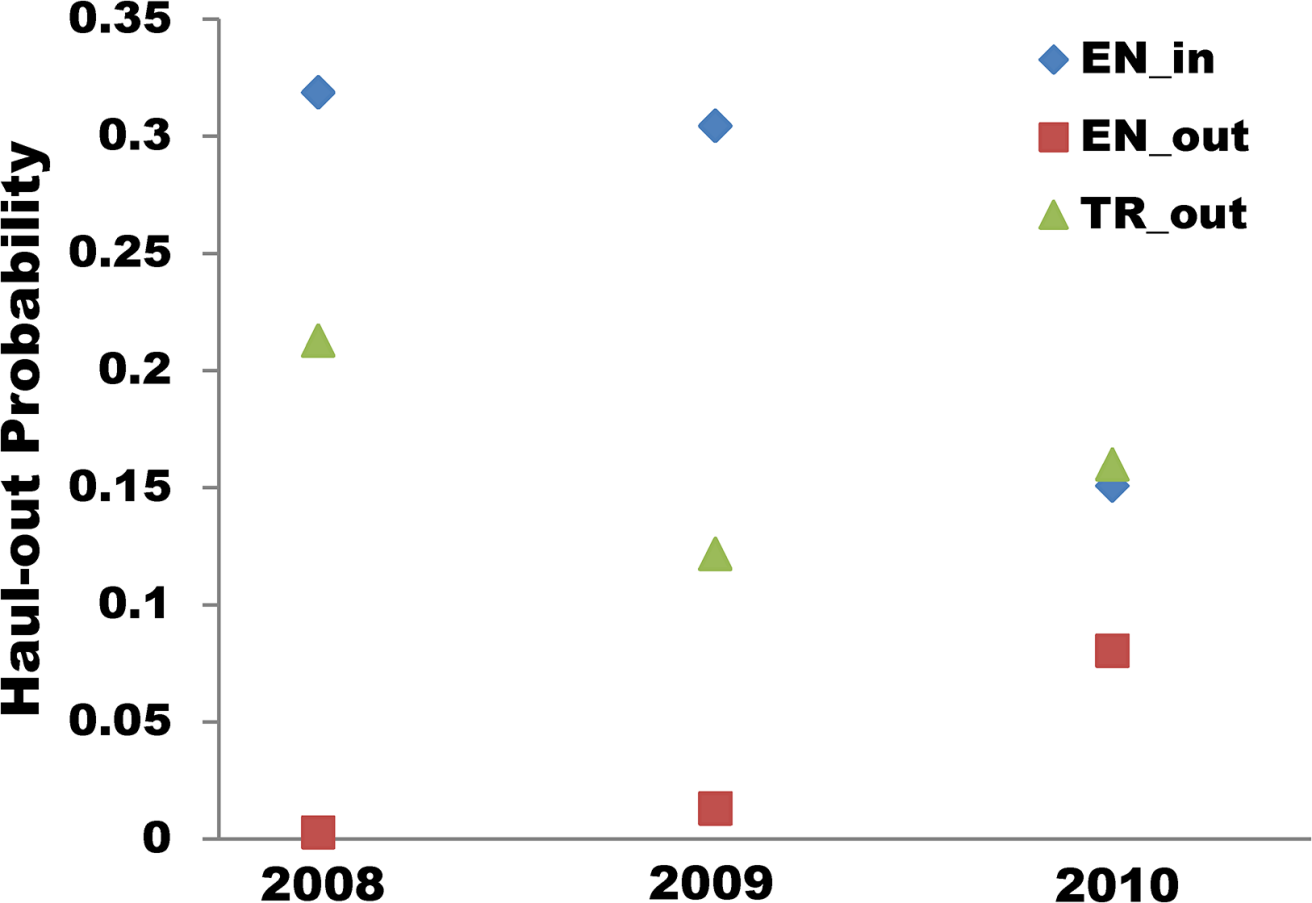
The probability that radio-tagged harbor seals were hauled out by inlet and year. Haul-out probability of radio-tagged seals when seals were present in inner Endicott (EN_in), outer Endicott (EN_out), and outer Tracy (TR_out) Arms in 2008, 2009, and 2010.

**Fig 8:**
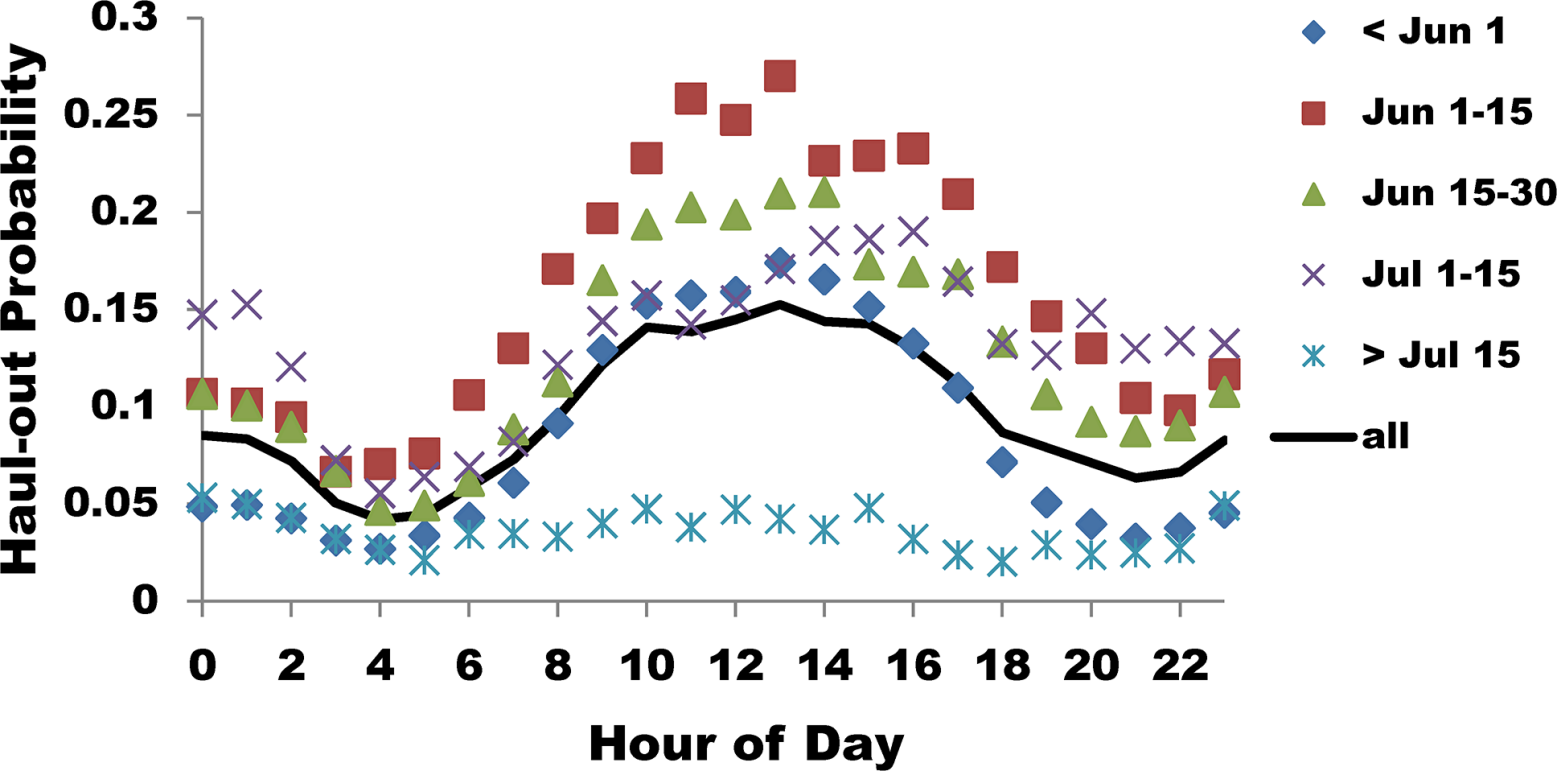
The probability that radio-tagged harbor seals were hauled out by hour of the day and season. Haul-out probability for radio-tagged harbor seals in Endicott and Tracy Arms combined, relative to hour of the day during each of five ~2 week seasons.

Haul-out probability was generally greatest at the inner Endicott location compared with the other two locations, but this varied somewhat with year (Table 4; Fig 7). Haul-out probability was lowest in season 5 (>15 Jul), which also had little hourly variation (Fig 8). Other seasons had substantial variation in hourly haul-out probability with highest probabilities during the day, lower in the evening, and lowest in the early morning. Of seasons showing this pattern, season 2 (early June) had the highest overall haul-out probabilities, followed by season 3 (late June; Fig 8).

**Fig 9:**
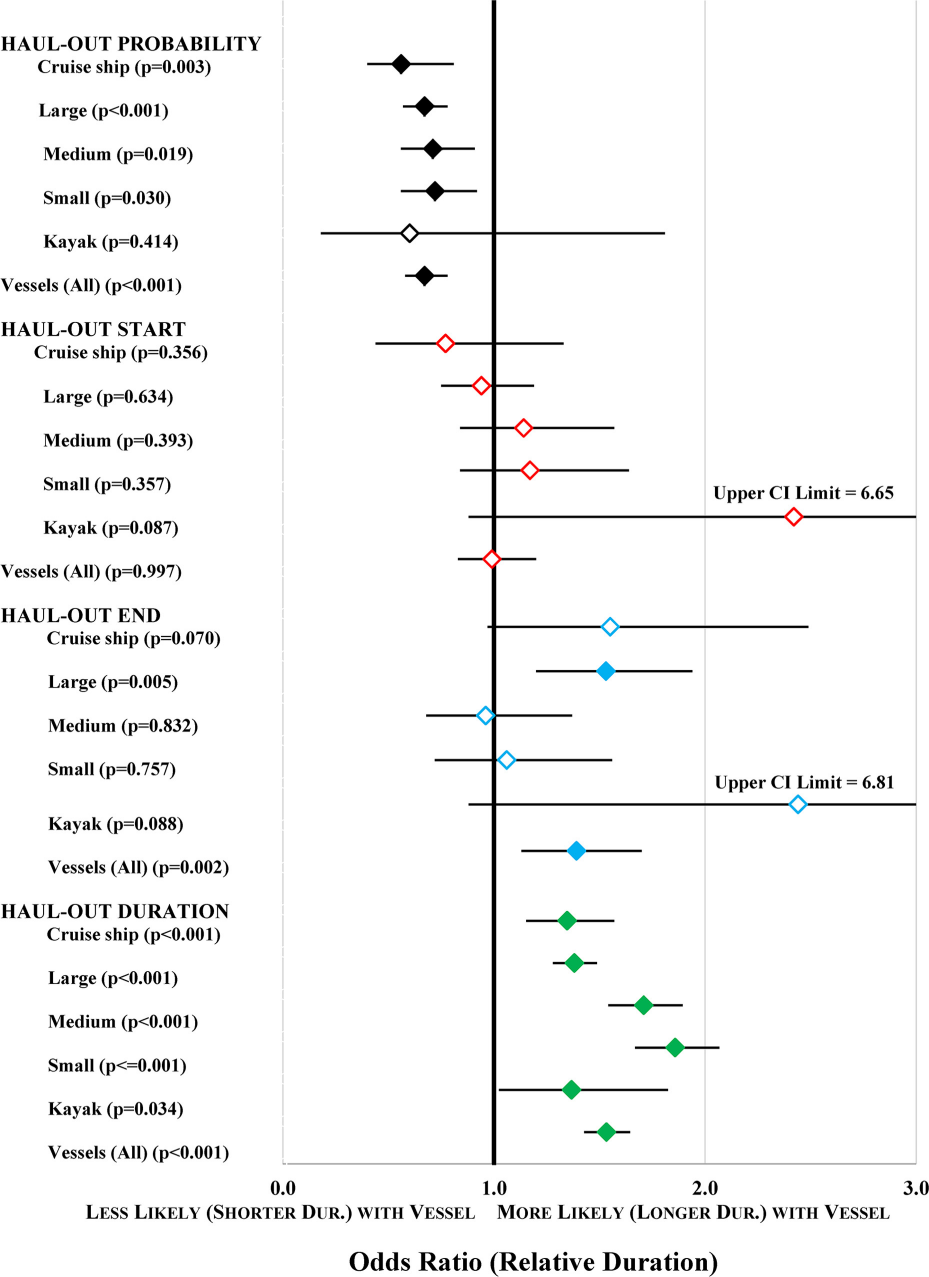
Summary of Vessel Effects on Seal Behavior. Estimates of haul-out probability, haul-out start, and haul-out end are odds ratios, which estimate the effects of vessel presence for each size category, relative to when there were no vessels of that size present. Estimates for durations are the geometric mean duration (dur.) of haulouts when a vessel was present relative to when there were no vessels. The observed effect of longer duration of haulouts in the presence of vessels is likely spurious, as a result of the confounding effects of the presence of numerous vessels during midday, at a time when the majority of seals haul out (Fig 8). Significant effects (i.e., factors where the CI does not cross 1 or no effect) have solid symbols.

The odds of a seal being hauled out were greatly reduced by the presence of a vessel (Table 4; Fig 9). Cruise ships had the strongest effect (i.e., seals had a lower probability of being hauled out when cruise ships were present), but the confidence intervals for the odds ratios for the various vessel types broadly overlapped (Table 4; Fig 9). Although the data suggest that kayak presence negatively affected haul-out probability, the effect is not significant due to imprecise estimates (small sample size) as a result of low detectability of kayaks among the icebergs when the photographs were inspected for vessel presence.

### Haul-out Duration

**Fig 10:**
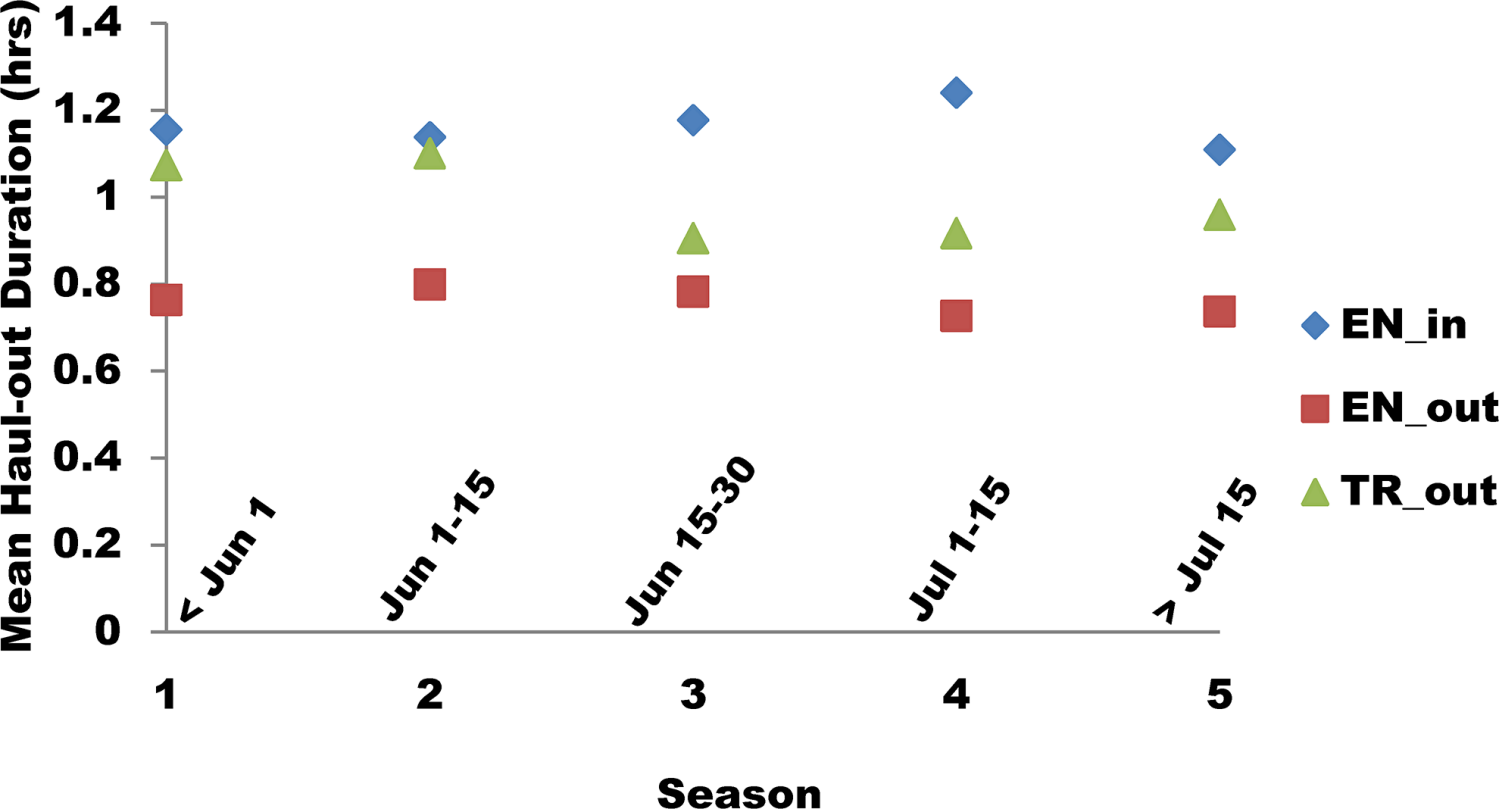
Haul-out duration for radio-tagged harbor seals by season and inlet. Geometric mean duration (in hours) of haulouts for seals in Endicott and Tracy Arms during each ~2 week season.

**Fig 11:**
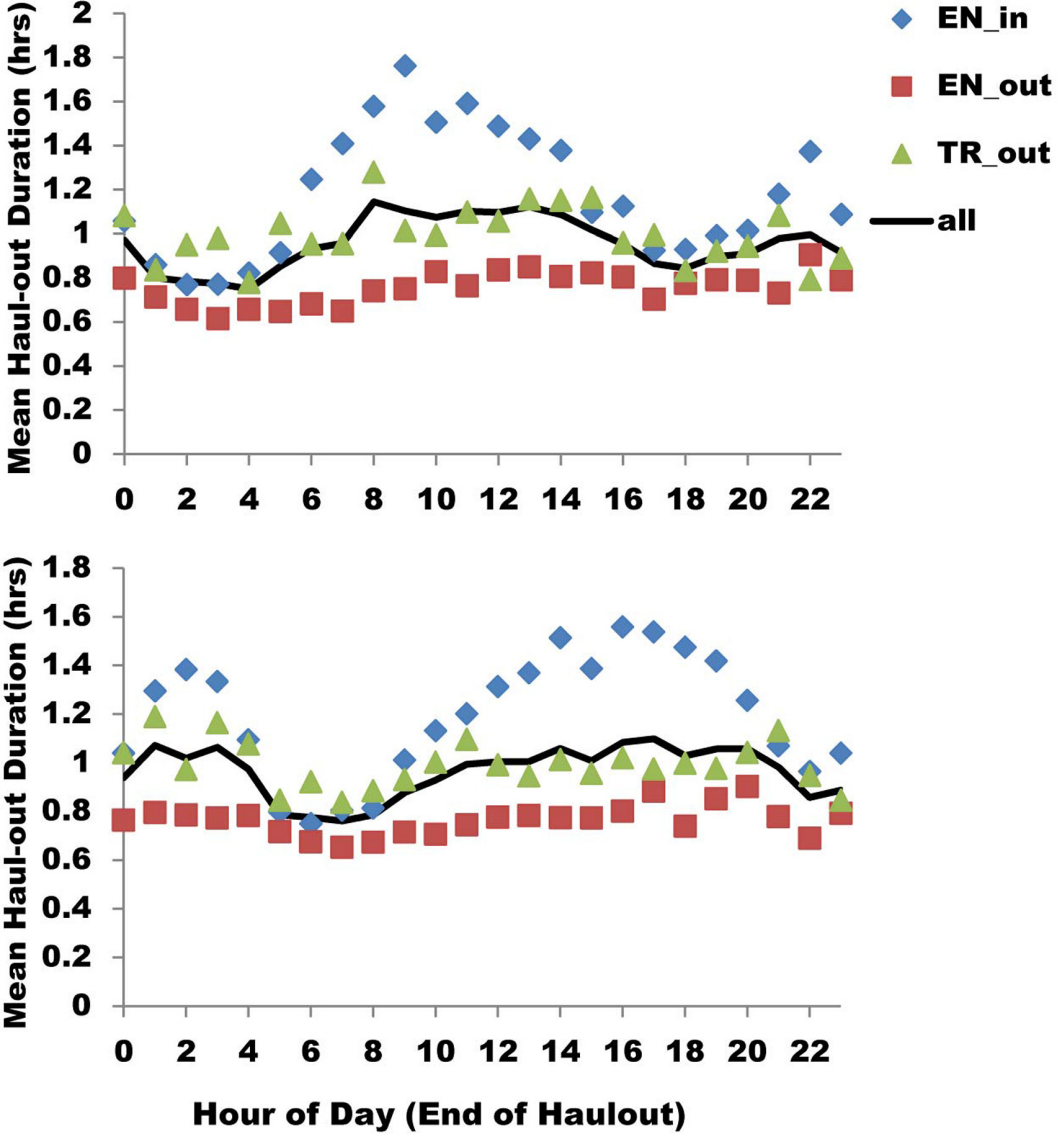
Haul-out duration for radio-tagged harbor seals in each inlet by start and end time. Top Panel: Geometric mean duration (in hours) of haulouts for radio-tagged harbor seals in inner Endicott (EN_in), outer Endicott (EN_out) and outer Tracy (TR_out) Arms relative to hour of the day at the start of the haulout. Bottom Panel: Average duration (in hours) of haulouts for radio-tagged harbor seals detected at each monitoring site relative to hour of the day when the haulout ended.

Geometric mean haul-out durations, similar to haul-out probabilities, were low for outer Endicott, high for inner Endicott, and intermediate for Tracy; mean durations in Tracy showed the most variation across seasons (Table 5; Fig 10). Mean haul-out durations were generally highest for bouts that started during the middle of the day and ended in the afternoon or evening (Fig 11). The daily pattern varied strongly among locations, with little hourly variation at outer Endicott, strong variation at inner Endicott, and Tracy had intermediate variation (Fig 11).

**Table 5:** Mean haul-out duration in relation to predictor variables.

Predictor	P-Value	Effect (95%CI)	Marginal Mean (hrs) (95% CI)
**Year**	<0.001		
2008			1.14 (1.04,1.24)
2009			0.94 (0.88,1.01)
2010			0.82 (0.76,0.87)
**Season**	0.477		
**Location**	<0.001		
Inner Endicott			1.16 (1.10,1.22)
Outer Endicott			0.76 (0.70,0.83)
Outer Tracy			0.99 (0.88,1.10)
Season*Location	0.128		
**Sex**	0.320		
Season*Sex	0.775		
**Hour at Start(hr_start)** ^**1**^	<0.001		Fig 11
Season* hr_start	<0.001		
Location* hr_start	<0.001		
Sex* hr_start	0.475		
**Hour at End (hr_end)** ^**1**^	<0.001		Fig 11
Season* hr_end	<0.001		
Location* hr_end	<0.001		
Sex* hr_end	0.318		
**Tidal Flow**	<0.001		
Falling			1.15 (1.07,1.24)
Rising			1.32 (1.22, 1.41)
Slack			0.89 (0.82,0.96)
**Ice**	<0.001		
Low			0.80 (0.73,0.88)
Medium			0.92 (0.83,1.01)
High			1.09 (0.98,1.21)
**Sky**	<0.001		
Clear			1.83 (1.57,2.13)
Partly Cloudy			1.11 (1.04,1.22)
Foggy			0.54 (0.48,0.61)
Overcast			0.67 (0.61,0.73)
**Precipitation**	0.001		
No			0.99 (0.91,1.08)
Yes			0.87 (0.79,0.95)
**Cloud Cover**	<0.001	1.011 (1.009,1.012)	
**Vessels** ^**2**^			
Cruise Ship = 0	<0.001		0.91 (0.85,0.97)
Cruise Ship> = 1			1.22 (1.04, 1.43)
Large = 0	<0.001		0.88 (0.83, 0.94)
Large> = 1			1.22 (1.11, 1.33)
Medium = 0	<0.001		0.89 (0.83, 0.95)
Medium> = 1			1.52 (1.36, 1.70)
Small = 0	<0.001		0.89 (0.84, 0.95)
Small> = 1			1.66 (1.48, 1.86)
Kayak = 0	0.034		0.92 (0.86, 0.98)
Kayak> = 1			1.26 (0.94, 1.68)
Vessels (all) = 0	<0.001		0.84 (0.79, 0.90)
Vessels (all)> = 1			1.29 (1.19, 1.41)

For continuous predictors (i.e., cloud cover) the effect is the change in haul-out duration for a 1 unit change in the predictor. Marginal means (i.e., SAS least-squares means; [41]) are the estimated mean duration of haul-out bout at each level of the categorical predictor variable, adjusted for other variables in the model.

^1^‘hr_start’ and ‘hr_end’ not used simultaneously.

^2^Results for binary vessel predictors (i.e., absence/presence); results for the binary vessel variables are easier to interpret, but show similar patterns to the continuous vessel predictor models.

Other factors that affected haul-out duration included tidal flow, ice cover, sky conditions, precipitation, and cloud cover (Table 5). Haul-out duration increased with high ice cover, clear skies, and no precipitation. Haul-out duration also increased with rising tides, indicating that seals may use directional current to assist in traveling to glacial habitat, but were unlikely to swim against the current to exit that habitat. Alternatively, incoming tides tend to push ice toward the heads of the inlets, which may serve to pack together and stabilize icebergs, in addition to making it more difficult for vessels to navigate within the ice. These effects could potentially increase the suitability of the icebergs as haul-out substrate. In contrast, outgoing tides can disperse ice which may move haul-out substrate to less desirable locations, reduce the availability, and/or make the ice conditions more navigable for vessels, facilitating closer approaches to seals.

Contrary to the pattern observed for haul-out probability, in which vessel presence was associated with lower probability of being hauled out, haulout duration was longer in association with the presence of vessels (Fig 9). Although the models attempted to correct for hour of the day, we believe the effect of longer haulouts with vessel presence is spurious, as a result of the confounding effects of the presence of numerous vessels during midday, at a time when the majority of seals haul out (Fig 8).

### Haul-out Start

The best predictors of an observation being at the start of a haulout (compared to a mid-haul-out observation) were hour of the day, precipitation, sky condition, and cloud cover (Table 6). These weather variables were strongly related, all indicating that haul-out starts were more likely with increasing cloud cover or fog, and with precipitation. We found little evidence that vessel presence was associated with the start of a haul-out bout (Fig 9).

**Table 6:** Predictors related to the probability of an observation being a ‘start-of-haulout’ observation.

Predictor	P-Value	Odds Ratio (95%CI)
**Hour of Day**	<0.001	
**Tidal Flow**	0.578	
**Precipitation** ^**1**^ ^,^ ^**2**^	<0.001	1.43 (1.24,1.66)
**Sky Condition** ^**1**^ ^,^ ^**3**^	<0.001	
Partly Cloudy	0.223	1.14 (0.92,1.41)
Overcast	0.001	1.37 (1.14,1.65)
Foggy	<0.001	1.76 (1.30,2.38)
**Cloud Cover (%)** ^**1**^ ^,^ ^**4**^	<0.001	1.003 (1.001,1.005)
**Ice Cover**	0.110	
**Vessels** ^**5**^		
Cruise Ship (184)	0.356	
Large (691)	0.634	
Medium (355)	0.393	
Small (131)	0.357	
Kayak (16)	0.087	2.42 (0.88,6.64)
Vessels (all)	0.997	

Predictors of the probability of an observation being a ‘start-of-haulout’ observation were assessed compared to predictors consistent with ‘middle-of-haulout’ observations. Odds ratios are the relative odds of a seal being hauled out for one value of the covariate vs. another value [47]. Numbers in parentheses for vessel predictors are numbers of vessels.

^1^Precipitation, sky condition, and cloud cover are strongly related; results are for each variable singly.

^2^The odds ratio for precipitation is for ‘some’ relative to ‘none’.

^3^The odds ratios for sky conditions (‘Partly Cloudy’, ‘Overcast’, and ‘Foggy’) are relative to ‘Clear’.

^4^The odds ratio for Cloud Cover is the change for a 1% increase in cloud cover.

^5^Results for binary vessel predictors (i.e., absence/presence); results for the binary vessel variables are easier to interpret, but show similar patterns to the continuous vessel predictor models. Odds ratios for vessel predictors are the odds of a ‘start’ observation when a vessel is present, relative to when no vessel is present.

### Haul-out End

The best predictors of a haul-out-ending observation (compared to a mid-haul-out observation) were hour of the day, cloud cover, ice cover, and vessel presence (Table 7). End observations were associated with increasing cloud cover and low ice cover. The presence of large-sized vessels and all-vessels combined were strongly and significantly associated with the odds that the observation was an end observation (Fig 9). The point estimates for cruise ships and kayaks also were positively associated with haul-out ends, but likely because of smaller sample sizes, the variances are higher and hence the support for these vessel types is weaker (p = 0.070 and 0.088, respectively).

**Table 7:** Predictors related to the probability of an observation being an ‘end-of-haulout’ observation.

Predictor	P-Value	Odds Ratio (95%CI)
**Hour of Day**	<0.001	
**Tidal Flow**	0.158	
**Precipitation**	0.261	
**Sky Condition**	0.764	
**Cloud Cover (%)**	0.007	1.003 (1.001,1.005)
**Ice Cover**	<0.001	
Low	<0.001	1.77 (1.41,2.22)
Medium^1^		
High	0.984	1.06 (0.97,1.16)
**Vessels** ^**2**^		
Cruise Ship (184)	0.070	1.55 (0.97,2.49)
Large (691)	0.005	1.53 (1.20,1.94)
Medium (355)	0.832	
Small (131)	0.757	
Kayak (16)	0.088	2.44 (0.88,6.81)
Vessels (all)	0.002	1.39 (1.13,1.70)

Predictors of the probability of an observation being an ‘end-of-haulout’ observation were assessed compared to predictors consistent with ‘middle-of-haulout’ observations. Odds ratios are the relative odds of a seal being hauled out for one value of the covariate vs. another value [47]. Numbers in parentheses for vessel predictors are numbers of vessels.

^1^Odds ratios for ‘Low’ and ‘High’ ice are relative to ‘Medium’ ice.

^2^Results for binary vessel predictors (i.e., absence/presence); results for the binary vessel variables are easier to interpret, but show similar patterns to the continuous vessel predictor models. Odds ratios for vessel predictors are the odds of an ‘end’ observation when a vessel is present, relative to when no vessel is present.

### Covariate Effects Relative to Time of Day

The probability of specific environmental conditions occurring during different blocks of time varied (Table 8). Although numerous significant differences were noted as a result of our large sample sizes, the differences in probability levels among time blocks were relatively small. With respect to those covariates that affected haul-out behavior of seals, percent of ice cover did not have a strong pattern; the highest concentration of ice tended to occur in the afternoon, however the probability was not significantly different than evening or late morning. There was no variation in occurrence of medium ice coverage and only weak patterns for low ice coverage; low ice had similar probabilities of occurring in early/late morning, and evening. Partly cloudy was the only sky condition that was different across all time blocks, with a higher probability of occurring in the afternoon. There was a higher probability of fog in the early morning, followed by the evening; late morning and afternoon were not different. The occurrence of precipitation did not have a strong pattern; the highest probability of rain was in the evening which did not differ from the afternoon, afternoon and early morning were not different, and rain had the lowest probability of occurring during late morning. Cloud cover was more likely to be greater in the evening and early morning, followed by afternoon and late morning. Vessels had the highest probability of being present in the late morning (mean probability of occurrence 0.57) when seals were generally hauled out, compared to a mean probability of 0.22 of occurring in the afternoon when seals were generally ending haulouts, and mean of 0.11 and 0.06 probability of occurring in the early morning or evening, respectively (Table 8).

**Table 8:** Comparison of covariates by time-of-day categories.

Variable		Early morning	Late morning	Afternoon	Evening
**Sky**	**Clear**	0.17 (0.15, 0.20) ab	0.20 (0.18, 0.22) a	0.16 (0.14, 0.18) b	0.18 (0.16, 0.20) ab
	**Partly Cloudy**	0.20 (0.18, 0.22) d	0.26 (0.24, 0.27) b	0.29 (0.27, 0.32) a	0.22 (0.21, 0.24) c
	**Foggy**	0.13 (0.12, 0.15) a	0.07 (0.06, 0.08) c	0.07 (0.06, 0.08) c	0.10 (0.09, 0.12) b
	**Overcast**	0.49 (0.47, 0.52) a	0.48 (0.46, 0.50) a	0.48 (0.45, 0.51) a	0.49 (0.47, 0.51) a
**Precip**	**Yes**	0.29 (0.27, 0.31) b	0.25 (0.23, 0.27) c	0.31 (0.29, 0.35) ab	0.33 (0.31, 0.36) a
**Tide**	**Rising**	0.31 (0.30, 0.33) c	0.42 (0.40, 0.44) a	0.43 (0.41, 0.45) a	0.39 (0.37, 0.40) b
	**Falling**	0.45 (0.43, 0.47) a	0.34 (0.33, 0.36) b	0.32 (0.30, 0.33) c	0.36 (0.34, 0.37) b
	**Slack**	0.24 (0.23, 0.24) b	0.24 (0.23, 0.24) b	0.25 (0.25, 0.26) a	0.26 (0.25, 0.26) a
**Ice**	**Low**	0.47 (0.44, 0.51) a	0.43 (0.40, 0.46) ab	0.41 (0.38, 0.44) b	0.44 (0.41, 0.47) ab
	**Medium**	0.33 (0.31, 0.36) a	0.36 (0.33, 0.39) a	0.36 (0.34, 0.39) a	0.35 (0.33, 0.38) a
	**High**	0.15 (0.14, 0.18) b	0.18 (0.16, 0.20) ab	0.20 (0.18, 0.22) a	0.17 (0.15, 0.20) ab
**Cloud Cover**	**Percent**	71.2 (68.0, 74.3) a	66.5 (63.4, 69.6) c	68.8 (65.7, 71.9) b	71.3 (68.2, 74.4) a
**Vessels**	**Presence**	0.11 (0.07, 0.15) c	0.57 (0.53, 0.61) a	0.22 (0.18, 0.27) b	0.06 (0.02, 0.11) d

Time categories: Early Morning = 0500–0859; Late Morning = 0900–1259; Afternoon = 1300–1659; Evening = 1700–2059. Ice coverage (Ice): Low (<33%), Medium (33–66%), and High (>66%). The binary categories of Precipitation (Precip) and Vessels evaluate when precipitation was occurring (i.e., Precip = yes), or vessels were present. Estimates for categorical variables are probabilities that the observation is in that category; estimates for continuous variables (i.e., cloud cover and vessels) are means. Estimates within rows with the same letter are not statistically different.

## Discussion

Our research monitored background vessel activity in the general area where harbor seals haul out on icebergs, along with other variables that may influence haul-out behavior of seals, including temporal factors, weather, ice availability, and tidal flow. Seals showed high site fidelity to the Arm in which they were captured (Endicott), although there was some movement between Arms, as well as movement out of glacial habitats. There was individual and seasonal variability, however on average there was a 50% probability that a seal, radio-tagged in glacial habitat, was in that habitat.

Seals started haul-out bouts in potentially suboptimal conditions (e.g., rain and fog). However, fog occurred more often during the early morning when seals were just beginning to haul out, so that association could be coincidence. Whether seals remained hauled out or ended a haulout was influenced by time of day, weather, ice availability, and vessels. Seals had a higher probability of being hauled out with greater ice availability and during the middle of the day, and were less likely to be hauled out if the weather was poor, there was less ice, or vessels were present. Cruise ships had the strongest negative effect; however, all vessels with the exception of kayaks negatively affected haul-out probability (Fig 9). The lack of an effect for kayaks is likely attributable to small sample size and imprecise estimates, as a result of low detection of kayaks by cameras.

Large-sized vessels and all-vessels combined were significant predictors of the end of a haul-out bout, along with increasing cloud cover and lower ice availability. Cruise ship presence was associated with the end of haul-out bouts, however the effect was not significant (p = 0.070), likely because there were fewer cruise ships present later in the afternoon when seals were most likely to end haulouts (Fig 11).

That ice cover did not have a strong pattern relative to time of the day when different ice concentrations occurred and yet was a significant predictor of seal behavior singles this variable out as highly influential. Similarly, the fact that the highest vessel presence occurred during the hours when most seals were likely to be hauled out (Fig 8), yet vessel presence was a significant predictor of reduced haul-out probability (Fig 9), is strong evidence that vessels were driving that response. For other behavioral responses, there is evidence that the overlap in timing of vessel presence and seal haulouts may be confounding factors to some extent. Large vessels and all-vessels combined were significant predictors of haul-out end; however, vessel presence was lower in the afternoon and haul-out probability began to decline midway through that time block, suggesting that hours of vessel presence and timing of normal haul-out behavior may be somewhat confounded. The significance of all vessel predictors as being positively associated with duration of haulouts is almost certainly attributable to the confounding effects of an overlap in timing of high haul-out probability and high vessel occurrence.

Although there are numerous observational studies published on harbor seal responses to approaching vessels, the majority are from observations of seals in terrestrial habitat. To our knowledge, only four other published studies (of unmarked seals) investigated vessel disturbance of seals in glacial habitat [1–4]; however, comparison of our results with results from these studies is complicated. Jansen et al. [1] focused solely on seal response to approaching cruise ships by conducting observations (from onboard those ships) of focal seals hauled out on icebergs. The study estimated the distance at which seals flushed relative to the approach angle of the vessel, group size on individual bergs, and seal type (mother, pup, or other). That study did not quantify ice availability, however Jansen et al. [4] assessed ice availability relative to seal numbers and distribution, but locations of seals and cruise ships were not mapped concurrently. Hoover-Miller et al. [2] observed seal-vessel interactions in an area not visited by cruise ships, divided vessel categories into motorized vs kayaks, and broadly assessed ice availability as navigable or too dense to allow access to seals and the glacier face. Their study [2] was not able to evaluate distance between seals and vessels, but did quantify numbers of seals present. Young et al. [3] observed all types of vessels from a land-based observation site in a National Park where vessel traffic is regulated, and considered the effects of vessel type and distance, ice availability, and numbers of seals present.

While those studies used different methodologies to observe the direct response of harbor seals to approaching vessels, our study can only evaluate the relationship between haul-out behavior and background information on the presence of vessels and other environmental factors that could influence habitat quality and seal behavior. Our cameras underestimated total vessel presence, thus our estimates of vessel effects are conservative. Furthermore, we have no means by which to establish whether seals in our study were, in fact, responding to the presence and approach of vessels. Nonetheless, our study provides a great deal of previously unavailable information on haul-out behavior of radio-tagged seals over time, when vessels are known to be in the area. Our data showed patterns similar to those reported in observational studies of seal-vessel interactions—seals had a lower probability of being hauled out and a higher probability of ending a haulout if vessels were present (Fig 9).

Similarities in our results and the results of those observational studies are evident despite differences in the types of vessels present (among studies and within our own study) and the distances theoretically maintained between those vessels and seals. To protect harbor seals, vessel activity in parts of Glacier Bay National Park (including the glacial habitat used by the majority of seals in the Park [6]) is prohibited during pupping [3], and Park regulations require that vessels maintain a distance of 463m (0.25 nautical miles) from seals. That approach distance is greater than the current guidelines for distance that vessel operators are requested to maintain when viewing harbor seals elsewhere. The National Marine Fisheries Service (NMFS), Division of Protected Resources, recommends that a distance of at least 91m (100yards) be maintained from marine mammals to help reduce negative impacts of vessels on species protected under the Marine Mammal Protection Act. The researchers in Glacier Bay [3] observed disturbance of harbor seals by vessels at distances >463m for all vessel types except kayaks. When vessels were noncompliant with Park regulations and approached to ≤463m, all vessel types including kayaks resulted in disturbances; however, cruise ships disturbed more seals per vessel than did other vessel types.

No specific vessel regulations beyond the NMFS guidelines are imposed for vessels entering Kenai Fjords National Park [2]. That study evaluated interactions of vessels approaching within 300m of harbor seals and observed disturbance in 28% of those observations. Kayaks resulted in more disturbances than did motorized vessels, not including cruise ships which did not enter the study area.

The USFS manages the TAFT Wilderness Area where our study took place; however, the USFS cannot impose regulations on activities in marine waters, and vessel traffic in TAFT has shown a steady increase over the last decade (USFS unpublished). In addition to being cognizant of the NMFS viewing guidelines, many of the tourism vessel operators visiting TAFT voluntarily participate in a WBMP agreement (described in the methods). Cruise ships generally visit Tracy Arm if ice, visibility, and other cruise ship traffic are amenable to entry into the narrow inlet, while smaller commercial tourism vessels visit Endicott Arm. This variation in types of vessel traffic may account for some of the variability we observed in haul-out probability between Arms and among years in our study; however, ice, vessel presence, and/or an interaction of ice availability and vessel numbers explains much of the variation.

All of the seals in our study were tagged in inner Endicott and, when seals were present in glacial habitat, they were most often located in inner Endicott, consistent with other studies in which seals showed fidelity to the glacial habitat in which they were captured [8, 32]. Although ice availability was a significant predictor of haul-out probability in our study, the reduction by half of haul-out probability in inner Endicott (from 30% in 2009 to 15% in 2010; Fig 7) cannot be explained by diminished ice availability; ice availability was similar in both years. Conditions in outer Endicott in 2010 could partially explain reduced haul-out probability in inner Endicott that year. More ice was available in outer Endicott in 2010, we had a greater number of telemetry detections at that site that year, and there was an increased probability of seals being hauled out (1.2% in 2009 compared to 8% in 2010). Greater ice availability in outer Endicott may have made the outer inlet a better choice for hauling out than inner Endicott, because seals would have had less distance to travel to external foraging grounds from there, if those seals leave glacial habitat to forage as has been noted in Glacier Bay [32]. Nonetheless, those modest changes in haul-out probability do not adequately explain the magnitude of reduction, by half, of the probability of seals hauling out in inner Endicott in 2010.

That reduction in haul-out probability is better explained by a substantial increase in the presence of vessels. Vessels tend to move steadily through outer Endicott to reach inner Endicott, where they linger. Previous research, observing seal response to vessels, indicates that seals are less likely to be disturbed if a vessel moves at a steady pace, compared to stopping or moving erratically [21, 22]. The percentage of observations of all vessels in inner Endicott (Fig 4) and the numbers of vessels, by vessel category (Table 3), were higher in 2010 than 2009, with the exception of cruise ships and kayaks, which showed the opposite pattern. Because cruise ships are large and move slowly, they presumably have the highest likelihood of being detected by cameras, thus comparisons of cruise ship data between years is reasonably consistent. Kayaks, on the other hand, were generally not detected by the cameras. Our observation of higher kayak activity in 2009 compared to 2010 is a result of observations obtained from a shore-based camp maintained near the face of the glacier for 4 weeks in 2009, while no camp was present in 2010; thus, kayak presence is not adequately represented in 2010.

In 2010 in inner Endicott, 1–2 large vessels were known to be present for a total time of approximately 500 hours and ≥1 small vessels were present for >700 hours. Our data do not allow an evaluation of whether small vessels were associated with the large vessels that were present at the same time, thus we cannot determine whether the reduction in haul-out probability observed in 2010 was at least partially due to an additive effect for vessels. Nonetheless, the potential for an additive effect is high when large vessels deploy multiple smaller vessels to allow passengers to travel among the icebergs for a closer view of the glacier face and hauled-out seals. The fact that presence of large vessels was a significant predictor of the end of a haul-out bout, as was the effect of all-vessels combined, may indicate that an additive effect was occurring. Alternatively, the presence of undetected kayaks or skiffs released from those large vessels may have been the proximate cause of the haul-out end.

Changes in haul-out probability across years in inner Endicott are best explained by changes in vessel activity. In contrast, in Tracy Arm, ice availability and numbers of vessels were generally negatively related and had the effect expected if those factors interacted to result in conditions more conducive to hauling out. Compared to 2009, haul-out probability increased in Tracy Arm in 2010, concurrent with a marked increase in observations of high ice availability and almost no observations of low ice (Fig 3). In 95.2% percent of camera observations in 2010, there were no vessels of any type present in Tracy Arm, compared with 81% of observations with no vessels in 2009. Similarly, there were no detections of >2 vessels in Tracy in 2010, whereas in 2009 there were many observations of >2 vessels. AIS data obtained from mid-May to the end of July in both years indicates that vessel traffic for AIS-equipped, large-sized vessels and all cruise ships increased in TAFT in 2010. Although AIS data indicated that more of those vessels traveled up Tracy Arm in 2010 (164) compared with 108 in 2009, our cameras detected only 11 of 74 cruise ships in Tracy in 2010, and 13 of 90 large vessels. Those camera-detected, large vessels may also have included non-AIS vessels, because not all large vessels are equipped with AIS. The low detection of AIS vessels relative to numbers known to be in Tracy Arm likely is because thick ice prevented those vessels from progressing far enough up the Arm to come within view of the camera. Thus, high ice availability apparently provided ample substrate for seals to haul out on with reduced potential for vessel disturbance.

The presence of cruise ships had a more pronounced negative effect on probability of being hauled out than did other vessel types in our study and cruise ships were weakly associated with ending a haulout. Our data do not provide an assessment of distances or direct interactions between seals and vessels. Jansen et al. [1] noted that incidences of cruise ship disturbance of harbor seals increased as distances between seals and ships decreased; seals approached at 100m were 25 times more likely to flush than seals at 500m distances. Young et al. [3] observed that cruise ships resulted in the highest incidence of disturbance compared to all other vessel types, with disturbance occurring at distances >500m. It is possible that the high disturbance response noted in that study may be attributable to a lack of habituation to cruise ships for seals in that area. While other vessel types are permitted access to glacial habitat in Glacier Bay beginning 1 July, cruise ships are not allowed to enter before September and entries averaged only one ship per day. Thus, an approaching cruise ship was a relatively novel experience for seals in glacial habitat in Glacier Bay. In contrast, throughout the tourism season, cruise ships enter our study area and Disenchantment Bay, where the Jansen et al. [1, 4] studies took place.

Young et al. [3] noted that, per vessel, cruise ships disturbed more seals than did kayaks. In contrast, Hoover-Miller et al. [2] reported a higher proportion of disturbances were caused by kayaks than by motorized vessels, not including cruise ships which do not visit the study area. Our study did not adequately measure kayak presence, thus we cannot compare the relative effects of cruise ships vs. kayaks in association with the probability of ending a haulout.

An interesting difference between results of our study and those of Young et al. [3] is the effect of ice availability on seal behavior. Our study detected a higher probability of being hauled out and longer duration of haulouts with greater ice availability, and low ice coverage was a significant predictor of ending a haulout. In contrast, Young et al. [3] reported that seals were more likely to be disturbed (i.e., end a haulout) when there was more ice. The abundance of seals in that study was also positively associated with the amount of ice, causing the authors to speculate that the association of greater ice availability with greater disturbance may be attributable to increased vigilance of seals. Differences in distances between seals and vessels did not confound their assessment of ice availability and disturbance events. Regardless of approach distances, on days with high ice coverage, they observed that seals were >3 times more likely to flush than when ice coverage was low. The authors suggested that this may be a result of disturbance of seals that were closer to approaching ships, causing other seals farther from the vessels to respond to the behavior of those disturbed seals by abandoning their icebergs in response to an unknown threat. They also speculated that, when ice was sparse, ships may have been able to move at a constant speed along a predictable course of travel, which may reduce disturbance in response to erratic vessel behavior, as was noted in other studies of seals in terrestrial habitat [21, 22]. Jansen et al. [4] reported that seals tended to haul out in areas with intermediate ice availability, rather than in dense or sparse ice. The authors speculated that seals may avoid dense ice due to difficulties in finding open areas to surface for breathing or searching for aggregates of hauled-out seals.

Numerous studies have assessed vessel disturbance and advocated for mitigation to reduce vessel effects on harbor seals [1–3, 21, 24, 42]. Because seals in Alaska use glacial habitat seasonally, for pupping and molting [8], and tidewater glaciers host disproportionately large numbers of mothers and pups [5, 6], mitigation measures may be especially important to establish for vessels entering glacial habitats during those critical life-history periods.

Harbor seals appear to require a minimum (threshold) amount of haul-out time and have demonstrated compensatory behavior by temporarily increasing haul-out time after being deprived of the opportunity to haul out for a week [43]. Undisturbed resting time is especially important during the pupping and lactation periods [1] and during the energetically expensive molt [26]. Harbor seals lactate for only 3–5 weeks [44, 45] and pups weaned at lower weights have a lower probability of surviving their first year [46]. Therefore, frequent disturbance during the lactation period could reduce nursing time and compromise nutritional intake [32], and potentially endanger pup survival [46]. During molt, harbor seals spend more time hauled out and their skin temperature is increased during hair regrowth. The heat loss that occurs as a result of increased skin temperature—a physiological response that occurs naturally during molt—approximately doubles a seal’s resting metabolic rate [26]. When approaching vessels force seals to enter the water while their skin temperatures are elevated, their energetic costs likely increase in excess of the normal costs of the molting process, and repeated disturbances during the molt season could potentially prolong molt duration [26]. Forced entry into the icy waters of glacial habitat may exact even greater thermoregulatory costs [1] than in non-glacial habitat.

## Conclusions and Potential Mitigation

Glacial habitat is important pupping habitat for harbor seals in Alaska and provides constantly available substrate to haul out on during their short lactation period and during molt [32]. Mitigation to reduce the potential for vessel disturbance of harbor seals in glacial habitat may be especially important during these critical life-history phases. Ours is the first study to provide empirical data on the haul-out behavior of large numbers of individually-marked harbor seals in glacial habitat over a period of several months, while tourism vessels were present in that habitat. We assessed background levels of vessel traffic using time-lapse photographs, which likely underestimated vessel presence, and we examined haul-out behavior relative to vessel presence and other environmental and temporal factors. Our data provide information on timing and length of haulouts under the conditions we evaluated and revealed that vessel presence negatively affected a seal’s probability of being hauled out and was associated with ending a haulout. Our data do not confirm direct interactions or proximity of seals and vessels, thus results from other studies that observed direct seal-vessel interactions can be used in conjunction with our data on haul-out behavior of seals in the presence of vessel traffic. Collectively, those studies can serve to better inform management decisions related to minimizing the impact of tourism vessels on harbor seals, while concurrently allowing for tourism activities in glacial habitat.

Our results, derived from investigating haul-out behavior of >100 seals over 3 years, noted that haul-out duration was longest in the middle of the day and in association with greater ice availability, clear skies, an incoming tide, and no precipitation. The longest haul-out bouts generally ended in the late afternoon or evening. Seals were most likely to be hauled out between 08:00 and 18:00 (Fig 8) during most of the dates monitored, and haulouts initiated between 07:00 and 15:00 and ending between 14:00 and 19:00 (Fig 11) had the longest durations.

We believe that vessels and seals can coexist, however, we recommend that precautions be taken to minimize disturbance. We suggest that the potential for vessel disturbances of harbor seals could be reduced if, to the extent possible, the majority of vessel visits occurred before or after the hours of 08:00–17:00 or, less optimally, 09:00–16:00. We recognize that vessels that offer daily excursions, such as those to TAFT from Juneau or from other ports in Alaska to nearby glaciers, cannot avoid those midday hours. Other tourism vessels with extended itineraries that may include overnights stays could explore other scenic spots in the area during prime haul-out hours. The beauty of the glaciers could be enjoyed in the enhanced light (for photographs) of mornings and evenings, during the extended summer-daylight hours in Alaska.

A limit on the numbers of vessels entering glacial habitat during peak haul-out times could reduce disturbance and facilitate longer resting times for seals, especially on days with good weather and greater ice availability. Large-sized vessels that deploy kayaks and inflatables have the highest collective potential for disturbance. Vessel operators should choose their paths among the icebergs carefully and be vigilant for hauled-out seals. Observations by other researchers of seal response to vessels [1, 21, 22] revealed that seals are less likely to be disturbed when vessels move in a predictable manner (i.e., avoiding starts and stops), and when moving along a course parallel to or away from hauled-out seals. Vessel operators should take special care to avoid disturbing seals during pupping season, beginning in mid-May through the month of June, when the probability of seals hauling out is also highest.

Existing published data can be useful in establishing mitigation measures to reduce the potential for vessel disturbance of harbor seals. Indeed, in 2015, NMFS will release new guidelines designed to minimize disturbance by vessels visiting glacial habitat in Alaska. The guidelines are based on results from multiple research projects. Additional research is warranted, however, to estimate the frequency at which individual seals are disturbed by vessels, as well as assessing the potential implications of vessel disturbance with respect to the energetic costs of those disturbances and the potential for negative effects on fitness.

## Supporting Information

S1 Data File Seal ID telemetry summary.xls. Data file includes: Seal ID, sex, age, capture location and date, min/max and elapsed days of radio tracking.
(XLS)

S2 Data File TAFT_Tides_Ice2008-2010.xlsx. Data file includes: tide, weather data, and ice coverage for each telemetry station in each year.
(XLSX)

S3 Data File Vessel presence Times.xlsx. Data file includes: date, vessel type and time, weather, and cloud cover by location.
(XLSX)

S4 Data File VHFgood data.xlsx. Data file shows all VHF data (filtered to retain only good data). Includes date, time, seal ID, location, and transmitter type by year and telemetry station.
(XLSX)
